# Biomimetic Scaffolds with Dual Gradients of Biological Effectors for Tendon‐to‐Bone Repair

**DOI:** 10.1002/adhm.202503171

**Published:** 2025-07-15

**Authors:** Min Hao, Yidan Chen, Stavros Thomopoulos, Younan Xia

**Affiliations:** ^1^ The Wallace H. Coulter Department of Biomedical Engineering Georgia Institute of Technology and Emory University Atlanta GA 30332 USA; ^2^ School of Materials Science and Engineering Georgia Institute of Technology Atlanta GA 30332 USA; ^3^ Department of Orthopedic Surgery and Department of Biomedical Engineering Columbia University New York NY 10032 USA; ^4^ School of Chemistry and Biochemistry Georgia Institute of Technology Atlanta GA 30332 USA

**Keywords:** graded differentiation, hedgehog agonists, hydroxyapatite nanorods, interfacial diffusion, stem cells

## Abstract

Due to the complexspatial gradients in composition and structure at the native tendon‐to‐bone attachment, it remains a clinical challenge to repair rotator cuff tears. Herein, we describe a biomimetic scaffold with dual gradients in osteogenic and tendon enthesis effectors to regulate the graded differentiation of stem cells for tendon‐to‐bone repair. Funnel‐shaped microchannels prompted stem cells to rapidly infiltrate into the scaffold to experience the dual gradients in biological effectors along the walls of each microchannel. The graded distributions of two different effectors—hydroxyapatite nanorods and hedgehog agonist—worked synergistically to promote the differentiation of stem cells into osteoblasts, chondrocytes, and tenocytes, reproducing the cell phenotypes present in the natural tendon enthesis. The new scaffold offers a versatile platform to address key requirements for successful repair/regeneration of the tendon‐to‐bone attachment while also presenting an innovative strategy for the repair of connective tissue interfaces in general.

## Graphical abstract

The authors have developed a biomimetic scaffold with dual gradients through a diffusion‐driven method. The scaffold features funnel‐shaped microchannels for cell infiltration, while the dual gradients synergetically induce the graded differentiation of mesenchymal stem cells into osteoblasts, chondrocytes, and tenocytes, allowing for a seamless transition in cell phenotype to match the native tendon enthesis.

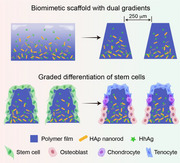

## 1 Introduction

Rotator cuff injury is a primary cause of shoulder joint dysfunction, and it is challenging to achieve successful and robust repair due to the difficulty in re‐attaching two tissues, tendon and bone, with dramatically different compositions, structures, and mechanical properties.^[^ ^1^ ^]^ The native tendon‐to‐bone attachment (the tendon enthesis) features a gradual spatial transition from tendon to non‐mineralized fibrocartilage, mineralized fibrocartilage, and bone, necessitating the arrangement of a spatial gradient in a diverse set of cell phenotypes.^[^ ^2^ ^]^ Importantly, there is no distinct boundary between adjacent regions of the tendon enthesis, resulting in a continuously‐graded distribution of extracellular matrix components, mechanical properties, and cell phenotypes.^[^ ^3^ ^]^ Despite advancements in surgical techniques, including open, mini‐open, and arthroscopic approaches,^[^ ^4^ ^]^ the high post‐operative failure rate of rotator cuff repair remains a significant clinical challenge. This issue primarily arises from the mechanical mismatch at the tendon‐to‐bone interface and the limited biological integration of the grafts utilized in these repair procedures.^[^ ^5^ ^]^ In general, the effective regeneration of the enthesis requires the establishment of a gradual and continuous transition in mechanical strength and cell phenotype to recapitulate the native insertion site. Tissue engineering strategies have shown promise in facilitating enthesis reconstruction by replicating its graded mechanical properties and guiding cellular responses. Various scaffold‐based approaches, including nanofiber mats,^[^ ^6^ ^]^ multiphasic scaffolds,^[^ ^7^ ^]^ polymer grafts,^[^ ^8^ ^]^ and decellularized tendon‐to‐bone grafts,^[^ ^9^ ^]^ have been explored, but all of them have limitations when translated to clinical use. For instance, while multiphasic scaffolds can replicate the hierarchical architecture of the enthesis, the abrupt phase transitions they introduce often lead to stress concentration at the interface and subsequent mechanical failure. Polymer grafts offer tunable mechanics but often lack precise spatial control over cell phenotype and require further optimization for effective integration. To address these limitations, we propose a dual‐graded polymer‐based scaffold that better mimics the native enthesis architecture and promotes a spatially controlled gradient of cell phenotypes.

Human‐derived mesenchymal stem cells (hMSCs), with their capacity for self‐renewal and multidirectional differentiation into osteoblasts, chondrocytes, and tenocytes, are promising candidates for achieving the necessary diverse cell phenotypes found at the native insertion.^[^ ^10^ ^]^ However, the effective use of hMSCs in this application is limited by their slow differentiation speed and the challenge of coordinating their graded differentiation.^[^ ^11^ ^]^ To address this issue, extracellular matrix proteins and minerals have been used to guide the differentiation of hMSCs into specific lineages. For instance, prior studies have demonstrated that hydroxyapatite (HAp), the key mineral found in bones, exhibits superior osteoinductivity.^[^ ^12^ ^]^ On the other hand, it is documented that regulating the hedgehog signaling pathway using antagonists or agonists could influence the chondrogenic and tenogenic potential of hMSCs.^[^ ^13^ ^]^ As a small‐molecule chemical agonist, hedgehog agonist 1.5 (HhAg) can regulate the Smoothened (Smo) receptor and initiate hedgehog signaling. However, direct injection of these small‐molecule effectors in vivo can result in uncontrolled diffusion, and the burst release from a carrier may compromise their effectiveness and result in adverse side effects.^[^ ^14^ ^]^ Therefore, developing a controlled release system for these biological effectors is critical for promoting the spatially controlled differentiation of stem cells. To this end, poly(ε‐caprolactone) (PCL) scaffolds incorporated with HAp nanostructures provides a versatile platform.^[^ ^15^, ^16^ ^]^ In particular, HAp nanorods with a uniform size distribution are synthesized in‐house for this study and utilized instead of conventional, irregular HAp powders from commercial vendors, as commercial powders form inhomogeneous PCL suspension. Additionally, the uniform size distribution of HAp nanorods contributes to their trackability during bone regeneration, as demonstrated by Li and coworkers, allowing for monitoring of morphological, compositional, and structural changes and potentially facilitating future in‐depth analysis of their in vivo functions.^[^ ^17^ ^]^ Here, we demonstrate that a combination of two biological effectors, HAp nanorods and HhAg, in spatially controlled gradients can regulate the graded differentiation of hMSCs, potentially augmenting graded transition zone regeneration at the tendon enthesis.

**Scheme 1:**
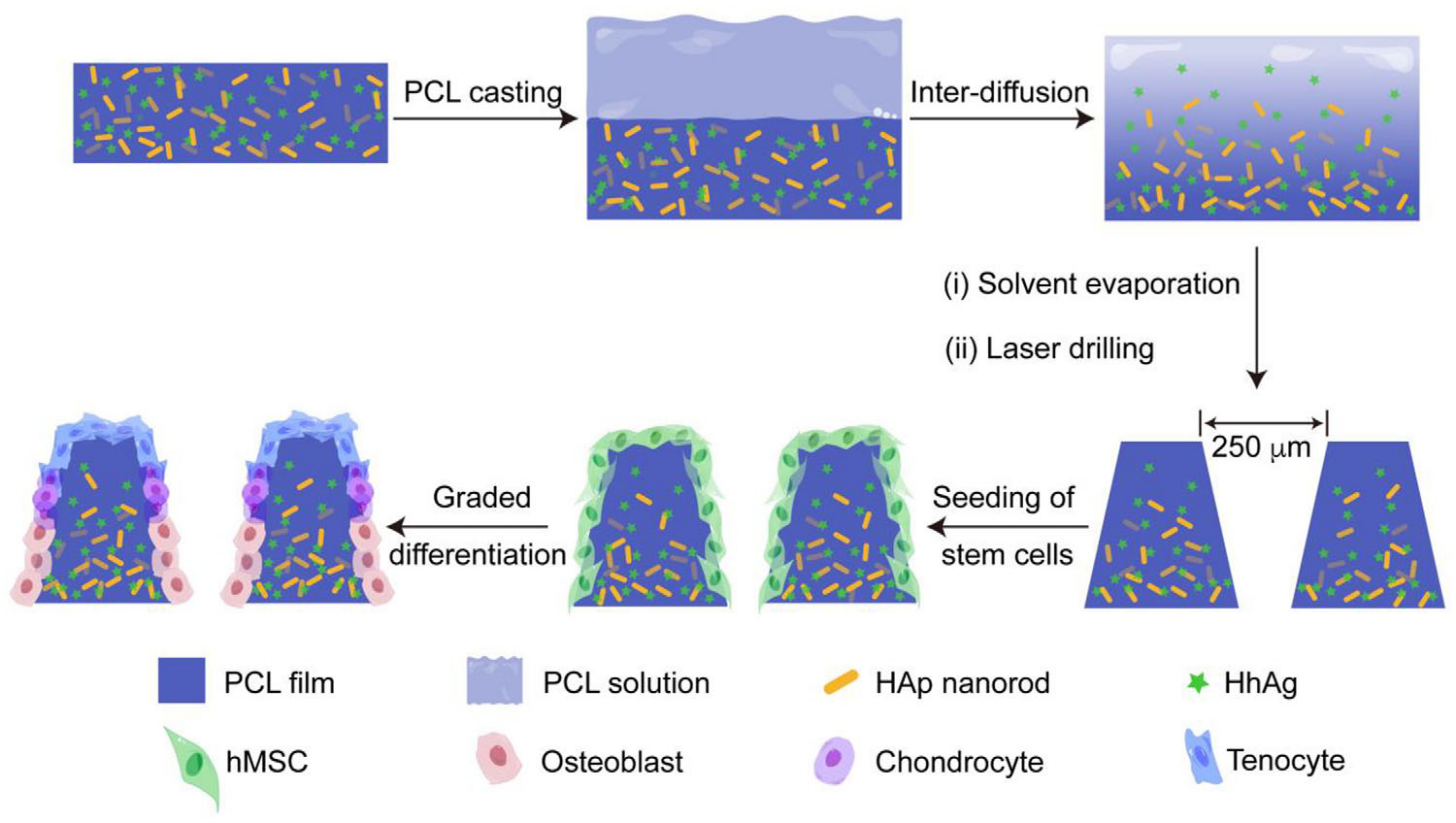
Schematic illustration depicting the fabrication of a biomimetic scaffold with graded distributions for both HAp nanorods and HhAg.

As shown in **Scheme** **1**, we used a diffusion‐based method to fabricate the biomimetic PCL scaffold with gradations in HAp and HhAg contents. Specifically, we started with the preparation of a PCL thin film containing uniformly distributed HAp nanorods and HhAg, followed by the addition of a PCL solution in 1,4‐dioxane onto the top of the film. Driven by diffusion, the distributions of HAp nanorods and HhAg evolved into gradations along the vertical direction of the film. After the solvent had evaporated, we used laser micromachining to create an ordered array of uniform, funnel‐shaped microchannels in each scaffold for cell infiltration and growth. The graded distributions in HAp and HhAg were instrumental to the graded differentiation of hMSCs into lineages necessary for the repair/regeneration of a robust tendon‐to‐bone insertion. In contrast to the strategies involving single effectors,^[^ ^18^ ^]^ we demonstrate the formation of a gradient with three different cell phenotypes. In addition, the HAp gradient induced a progressive mineralization transition to replicate the structure and composition of the native tendon enthesis. Taken together, the dual‐graded scaffold accommodates mechanical load transfer between compliant tendon and rigid bone, facilitating coordinated regeneration of the tendon‐to‐bone interface.

## 2 Results and Discussion

### 2.1 Fabrication and Characterizations of the Biomimetic Scaffold

**Figure 1:**
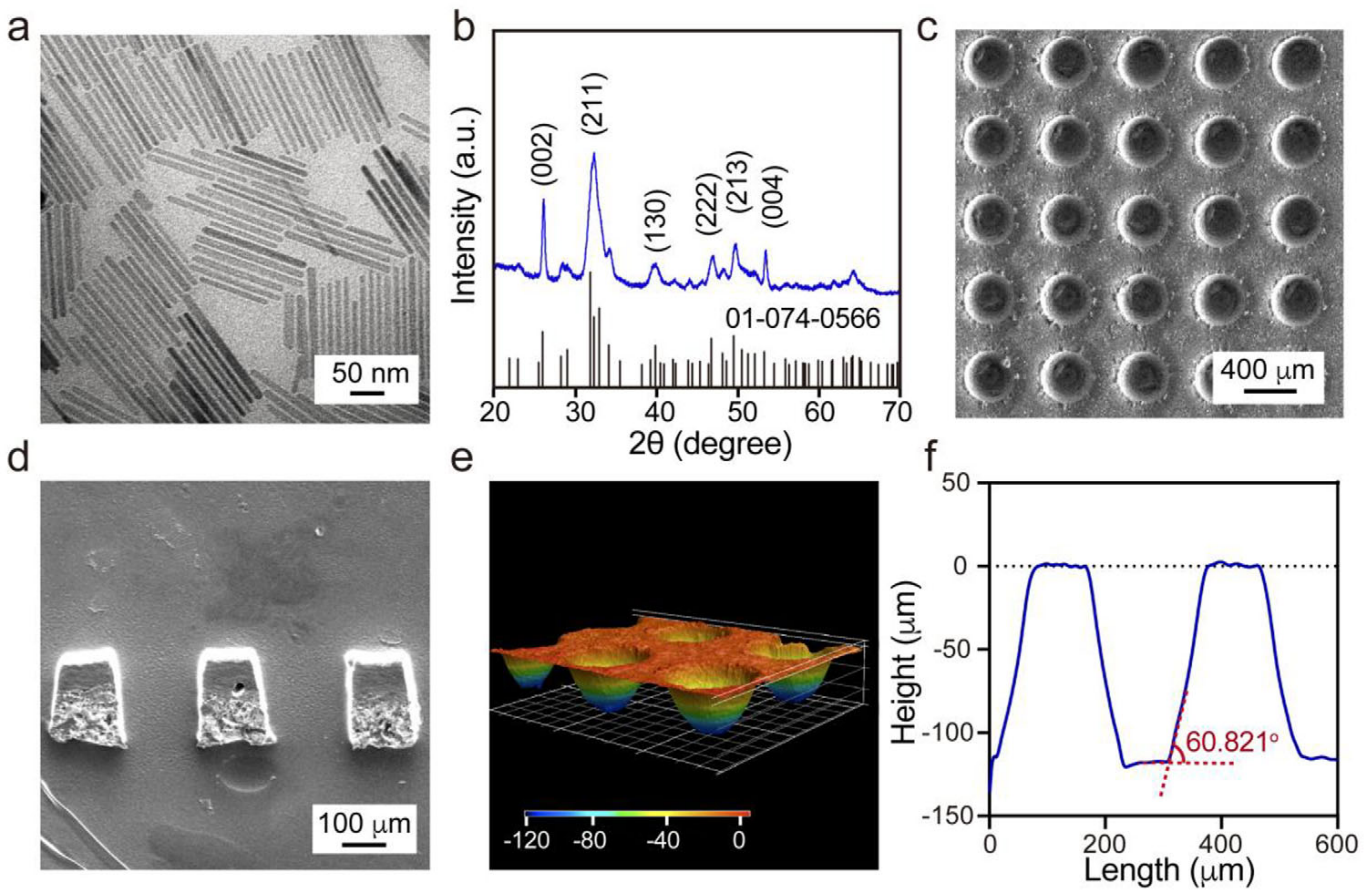
Characterizations of the HAp nanorods and HAp+HhAg biomimetic scaffolds. a) TEM image and b) XRD pattern of the HAp nanorods. c) Top view and d) cross‐sectional SEM images of the dual‐graded scaffold. e) 3D and f) cross‐sectional profile of the funnel‐shaped microchannels in the scaffold.

We first synthesized HAp nanorods by following a hydrothermal method.^[^ ^12^, ^17^ ^]^ As shown by the transmission electron microscopy (TEM) image in **Figure** **1a**, the nanorods had an average length of 130 ± 20 nm and an average width of 10 ± 2 nm. The X‐ray diffraction (XRD) pattern of these nanorods showed characteristic peaks consistent with the standard pattern (ICSD card no. 01‐074‐0566) of HAp with a hexagonal structure (Figure 1b). To investigate whether the morphology of the HAp nanorods changed during the embedding process, they were embedded in a PCL film for 3 days and then recovered by dissolving the PCL matrix with 1,4‐dioxane, followed by centrifugation. As shown in Figure S1 (Supporting Information), the recovered HAp nanorods shared a rod‐like morphology similar to that of the original nanorods, albeit the aspect ratio was somewhat reduced. It is important to note that this characterization reflects the post‐synthesis state of HAp nanorods within the scaffold. The complex and dynamic physiological environment in vivo could induce compositional and structural changes to the HAp nanorods after implantation for several months.^[^ ^17^ ^]^ We then prepared biomimetic scaffolds featuring graded distributions in both HAp and HhAg contents (denoted HAp+HhAg scaffolds) using a diffusion‐based method. As demonstrated in our previous work,^[^ ^19^ ^]^ funnel‐shaped microchannels can be fabricated through laser micromachining to facilitate cell infiltration and proliferation within the scaffolds. Figure 1c,d shows scanning electron microscopy (SEM) images of the top and cross‐section (cryo‐section) of the scaffold. The top view reveals an array of circular openings with a diameter of 245.3±10.0 µm, and the cross‐sectional image confirms the funnel shape of the microchannels. We also used an optical surface profiler to obtain the profiles of the microchannels (Figure 1e,f), revealing a funnel‐shaped profile with a slope angle of 60.8 ± 3°.

**Figure 2:**
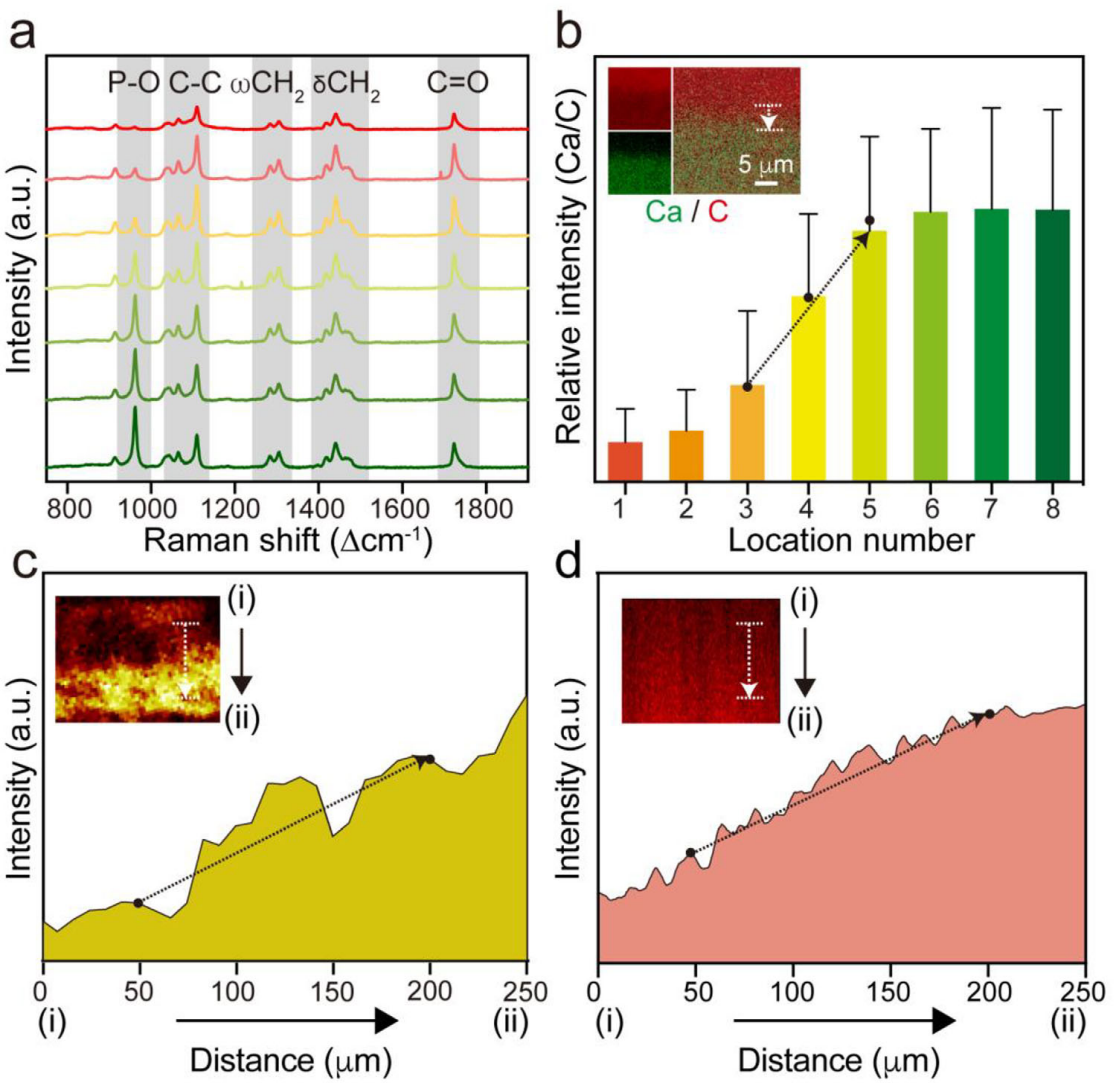
Graded distributions of HAp and HhAg in the biomimetic scaffold. a) Raman spectra recorded from different regions of the cross‐section of a scaffold, moving from the top (PCL only) to the bottom (enriched with HAp and HhAg). b) EDX mapping (inset) and a plot of the relative intensity between Ca and C in the graded region. c) ToF‐SIMS image (inset) and intensity plot based on Cl, moving from (i) PCL to (ii) HAp+HhAg/PCL zone. d) A plot of fluorescence intensity for a scaffold fabricated by replacing HhAg with RhB, moving from (i) PCL to (ii) HAp+RhB/PCL zone. The gray boxes in a) indicate the positions of the characteristic peaks. The regions marked by white arrows in the insets of b–d) correspond to the black arrows in the respective intensity plots. Data in b) are presented as mean ± standard deviation (SD) (N = 3).

Next, we examined the gradients of HAp and HhAg contents along the vertical direction of the scaffold. As shown by the Raman spectra in **Figure** **2a**, the intensity of P–O stretching at 960 cm^−1^ increased from the top to the bottom along the vertical direction, demonstrating a gradual increase in HAp content. Meanwhile, the peaks corresponding to PCL in terms of C─C stretching (1031–1109 cm^−1^), CH_2_ bending (1281–1306 cm^−1^), CH_2_ twisting (1418–1474 cm^−1^), and C═O stretching (1724 cm^−1^) slightly decreased in intensity. We calculated the ratio of the intensities of the P‐O stretching peak (960 cm^−1^) to the C═O stretching peak (1724 cm^−1^)^[^ ^20^ ^]^ and found that this value gradually increased from the top to the bottom of the scaffold (Figure S2, Supporting Information), confirming a graded distribution of HAp content along the vertical direction. In addition, considering the calcium (Ca)‐rich composition of HAp and the carbon (C)‐rich nature of PCL,^[^ ^19^ ^]^ we validated the graded distributions of Ca and C across the surface of a cryo‐sectioned scaffold through energy‐dispersive X‐ray spectroscopy (EDX) mapping (inset of Figure 2b). The content of Ca was the highest at the bottom of the scaffold, gradually decreasing toward the top. Conversely, the content of C showed a reverse trend. The location numbers 1 to 8 correspond to zones moving from the top to the bottom of the cross‐section of a scaffold. A semi‐quantitative analysis of the relative intensity of Ca and C (Figure 2b) indicated that the value at the bottom (location 8) of the scaffold was ca. 6.8‐fold higher than that at the top (location 1), trending in a gradual decrease from the bottom to the top. These data corroborated well with the Raman spectroscopy results, both demonstrating a graded distribution of HAp along the vertical direction of the scaffold. Given the critical role of a mechanical property gradient in alleviating stress concentrations at the tendon‐to‐bone interface and guiding cell behavior, we measured the Young's modulus along the vertical direction of the scaffold. The modulus showed a graded increase from ca. 0.62 to 1.39 GPa, which was consistent with the increasing HAp content along the vertical axis of the scaffold (Figure S3, Supporting Information). The result is in agreement with our previous finding in that the Young's modulus of an HAp/polymer composite positively correlated with the content of HAp in the polymer matrix.^[^ ^21^ ^]^ This gradient in mechanical strength, arising from the graded distribution of HAp, effectively bridges the mechanical mismatch between compliant tendon and rigid bone, and can potentially serve as a mechanical cue driving stem cell differentiation.

Leveraging the specificity of the chlorine (Cl) element in the HhAg molecule,^[^ ^22^ ^]^ we directly examined the distribution of HhAg along the vertical direction of the scaffold through time‐of‐flight secondary ion mass spectrometry (ToF‐SIMS), as shown in Figure 2c. A strong brightness indicates a high concentration of Cl (inset of Figure 2c). Notably, the concentration of Cl progressively increased from the top to the bottom, indicating a gradual increase of HhAg content along the vertical direction of the scaffold. We also utilized rhodamine B (RhB) as a fluorescent model compound and evaluated its distribution within the scaffold using confocal microscopy (Figure 2d).^[^ ^23^ ^]^ The fluorescence intensity of RhB gradually increased from the top to the bottom, demonstrating a graded distribution of this small molecule along the vertical direction of the scaffold. Altogether, these data demonstrated the presence of dual‐graded distributions of HAp and HhAg along the vertical direction of the scaffold.

### 2.2 Biocompatibility of the Biomimetic Scaffold

**Figure 3:**
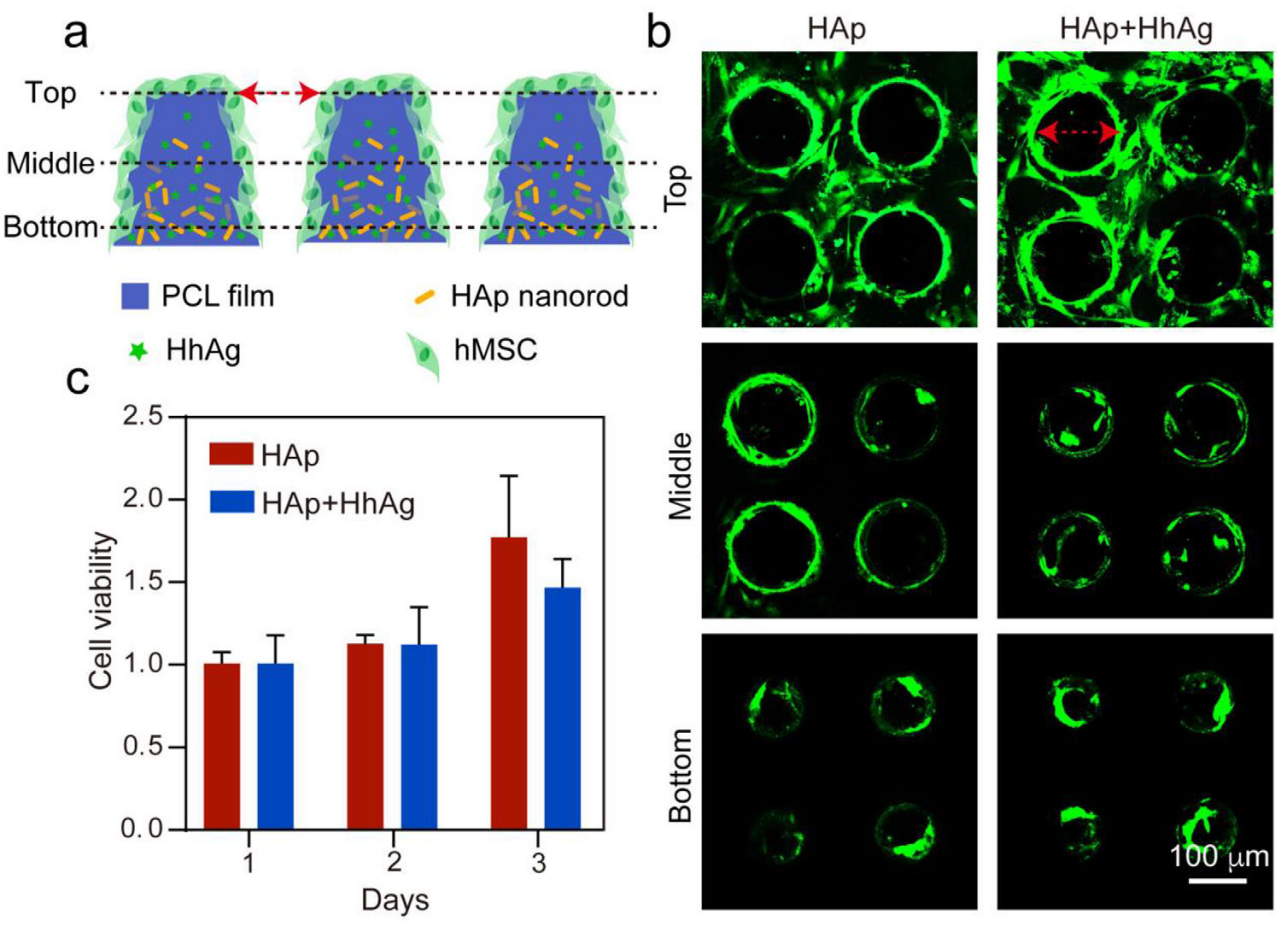
Biocompatibility of the biomimetic scaffolds. a) Schematic illustration depicting the analyzed sections of a scaffold. b) Fluorescence images of the scaffolds after live/dead staining of the hMSCs cultured for 24 h. The live and dead cells were stained green and red, respectively. The red bidirectional arrows in a) and b) indicate the same location: the funnel‐shaped microchannel. c) CCK8 assay of the hMSCs after culture with the scaffolds for 1, 2, and 3 days. The data are normalized to the HAp group on day 1 and presented as mean ± SD (N = 3).

The in vitro performance of the scaffold was evaluated by examining biocompatibility and cellular differentiation.^[^ ^24^ ^]^ We adopted both qualitative live/dead cell staining and a quantitative Cell Counting Kit‐8 (CCK8) assay to assess the biocompatibility of the scaffolds. Specifically, hMSCs were seeded on the HAp and HAp+HhAg scaffolds, respectively, and cultured for 24 h, followed by live/dead staining and visualization using confocal microscopy. We focused on three sections corresponding to the top, middle, and bottom of each scaffold (**Figure** **3a**). The vast majority of the cells in the three sections of both scaffolds showed a green stain, signifying their status as live cells, with only a few dead cells stained red (Figure 3b). This result was also evident in cross‐sectional and 3D Z‐stack confocal micrographs of the cell‐seeded scaffolds (Figure S4, Supporting Information). These data demonstrated that the scaffolds had excellent biocompatibility and that the inclusion of HhAg did not impair cell viability.

Subsequently, we performed a CCK8 assay to quantify cell viability after culturing the cells with the HAp and HAp+HhAg scaffolds, respectively, for 1, 2, and 3 days. In this assay, 2‐(2‐methoxy‐4‐nitrophenyl)‐3‐(4‐nitrophenyl)‐5‐(2,4‐disulfophenyl)‐2H‐tetrazolium sodium salt (WST‐8) was reduced by cellular dehydrogenases to generate orange formazan, thereby reflecting the viability of the cells.^[^ ^25^ ^]^ As shown in Figure 3c, the viability of cells in both samples gradually increased as the culture time was extended from 1, to 2, to 3 days, indicating robust cell proliferation in the scaffolds. On day 3, the cell viability in the HAp‐graded scaffold was higher than that in the HAp+HhAg‐graded scaffold. Since gradual differentiation of stem cells is typically accompanied by stable or decreased proliferation rates,^[^ ^26^ ^]^ this trend suggests that the cells in the HAp+HhAg‐graded scaffold had undergone differentiation. To investigate the viability of cells across different regions (top, middle, and bottom) of both HAp and HAp+HhAg scaffolds, scaffolds were cryo‐sectioned into three 50 µm‐thick sections, and then incubated with cells for 24 h. As shown in Figure S5 (Supporting Information), we observed consistently high cell viability throughout the vertical sections (top, middle, and bottom) of both scaffold types, indicating uniform biocompatibility. Taken together, both scaffolds demonstrated excellent biocompatibility, fulfilling a prerequisite for cell proliferation and growth.

### 2.3 Distribution and Morphology of hMSCs Seeded in the Scaffold

**Figure 4:**
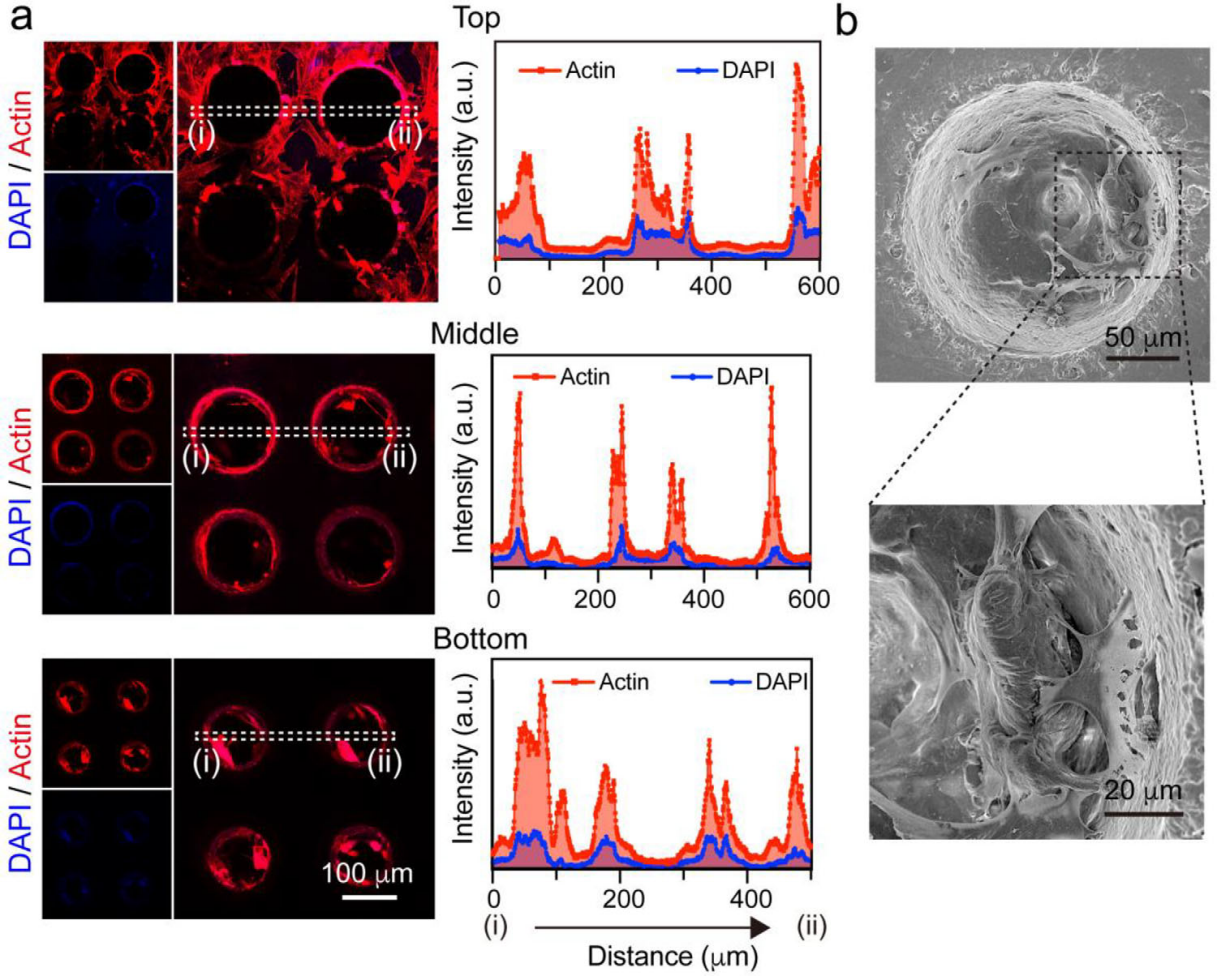
Distribution and morphology of the cells in the HAp+HhAg graded scaffolds. a) Fluorescence images (left) and fluorescence intensity distributions (right) of the hMSCs after culture with the scaffold for 24 h, followed by actin (red) and DAPI (blue) staining. The plots of fluorescence intensity correspond to the regions marked by dashed boxes. b) SEM images of the HAp+HhAg graded scaffold after being seeded with hMSCs and then cultured for 24 h.

Thorough distribution of cells within the scaffolds is necessary for the interfacial scaffold to bridge the healing tendon and bone. To examine cell distribution within the scaffold, we seeded hMSCs on HAp‐ and HAp+HhAg‐graded scaffolds and then cultured for 24 h, followed by actin and 4′, 6‐diamidino‐2‐phenylindole (DAPI) staining to visualize the cytoskeleton and nuclei, respectively, using confocal microscopy.^[^ ^27^ ^]^ Again, we focused on the top, middle, and bottom sections of each scaffold. As shown in **Figures** **4a** and S6a (Supporting Information), actin expression was mainly observed on the scaffold surface. The cells showed an extended, spreading‐out morphology, indicating excellent adherence to the scaffold surface. In the middle and bottom sections, actin expression was the strongest along the wall of the microchannels for both types of scaffolds. This observation was supported by changes in fluorescence intensity for actin and DAPI from location (i) to location (ii) in the confocal microscopy images. We further assessed the morphology and spreading of the cells within the microchannels using SEM. As shown in Figure 4b and Figure S6b (Supporting Information), the pseudopods of cells were able to anchor to the wall of the microchannel and spread across the surface. Taken together, these data confirmed that the cells infiltrated into the scaffold and proliferated along the microchannels of the scaffold, with the addition of HhAg having no adverse impact on cell spreading.

### 2.4 Effects of HAp and HhAg Effectors on Cell Differentiation and Signal Transduction

**Figure 5:**
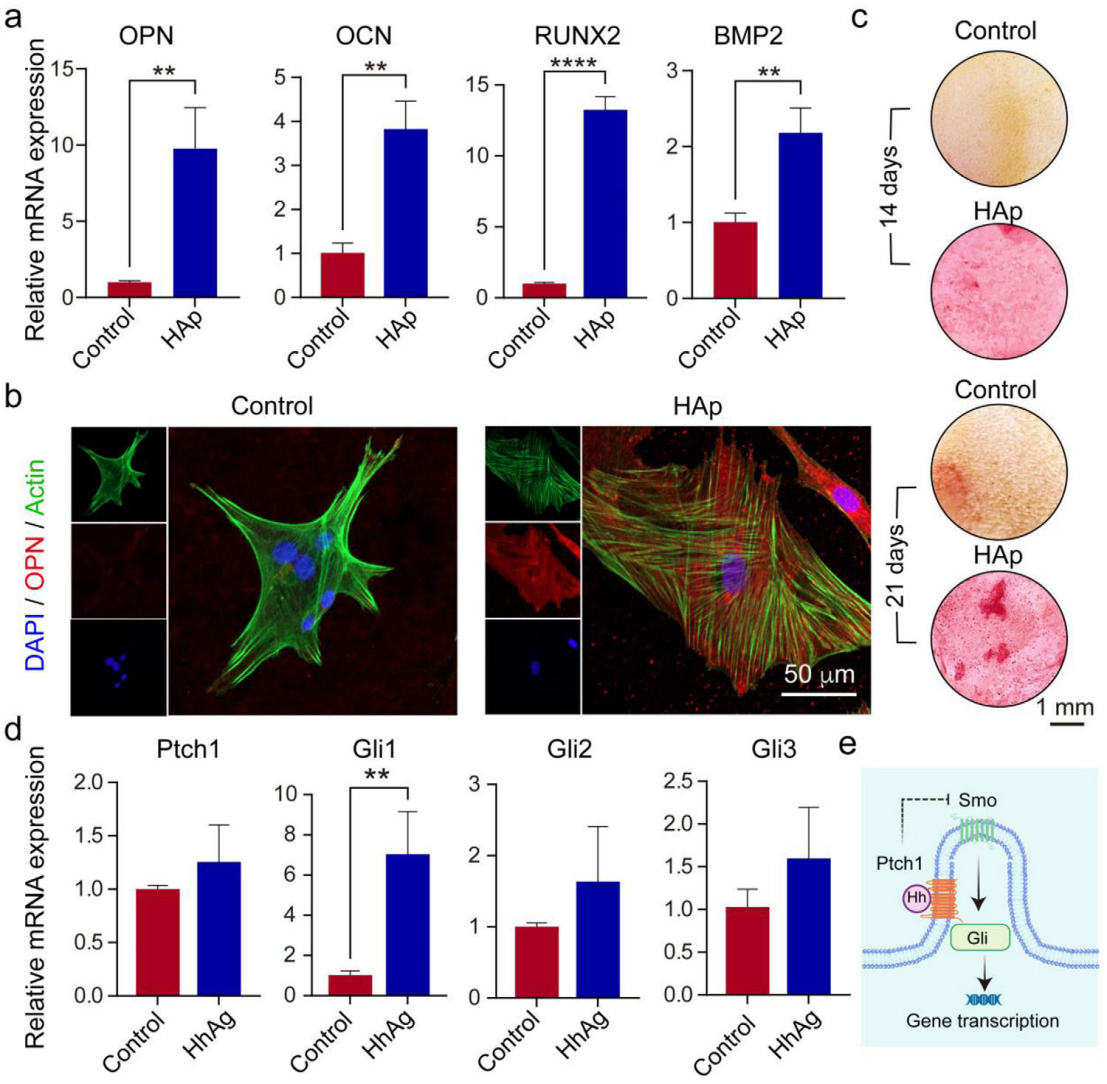
Regulation of HAp nanorods on cell osteogenic differentiation and HhAg on cell signal transduction. a) RT‐qPCR analysis of hMSCs culture on PCL plain films or PCL films containing HAp nanorods for 14 days. N = 3. b) Fluorescence micrographs of PCL plain films and PCL films containing HAp nanorods after actin (green), OPN (red), and DAPI (blue) staining of the hMSCs cultured for 21 days. c) Photographs of a PCL plain film and a PCL film containing HAp nanorods after alizarin red staining of the hMSCs cultured for 14 and 21 days. d) RT‐qPCR analysis of hMSCs culture on PCL plain films and PCL films containing HhAg for 14 days. N = 3. e) Schematic illustration of the transduction of Hh signaling. Statistical analysis for a) and d) was performed using a two‐tailed unpaired Student's t‐test (^**^ *p* < 0.01 and ^****^ *p* < 0.0001). Data are presented as mean ± SD.

To assess whether HAp nanorods promoted osteogenic differentiation, we cultured hMSCs on PCL plain films (control group) and PCL films with a constant concentration of HAp nanorods (HAp group) for 14 days, followed by examining the levels of osteoblast‐specific genes through reverse transcription‐polymerase chain reaction (RT‐qPCR) analysis (**Figure** **5a**). It is well‐documented that the bone morphogenetic protein 2 (BMP2) and Wnt signaling pathways are critical regulators in HAp‐induced osteogenic differentiation.^[^ ^28^ ^]^ HAp can upregulate the expression of BMPs and activate their receptors, thereby triggering the canonical BMP signaling pathway and the expression of osteogenic markers such as osteopontin (OPN) and osteocalcin (OCN).^[^ ^29^ ^]^ Additionally, the Wnt signaling pathway modulates Runt‐related transcription factor 2 (RUNX2), a key regulator of osteogenesis, ultimately driving the expression of osteogenic‐specific genes.^[^ ^30^, ^31^ ^]^ As such, we examined the expression of key genes, including OPN, OCN, RUNX2, and BMP2, that are associated with these pathways. The mRNA levels of OPN and OCN in the HAp group were ca. 9.8 and 3.8‐fold higher, respectively, than those in the control group. Similarly, the mRNA levels of RUNX2 and BMP2 increased by ca. 13.2 and 2.1‐fold relative to those of the control group, respectively. After 21 days of culture, the cells in both groups were stained with actin, OPN, and DAPI and then visualized using confocal microscopy (Figure 5b). The cells in the control group exhibited the spindle‐like morphology of MSCs, whereas those in the HAp group displayed a cuboidal morphology characteristic of osteoblasts. Moreover, the fluorescence intensity of OPN was higher in the HAp group than that of the control group. These data demonstrated that HAp nanorods were able to promote hMSCs differentiation into osteoblasts. Furthermore, we evaluated the mineralization activity of the cells in both groups using alizarin red staining at 14 and 21 days (Figure 5c).^[^ ^32^ ^]^ Notably, the cells in the HAp group exhibited a higher degree of mineralization compared to the control group, further confirming the ability of HAp nanorods to stimulate osteogenesis.

To evaluate the impact of HhAg on Hh signal transduction, we cultured cells on PCL films (control group) and PCL films containing a constant concentration of HhAg (HhAg group) for 14 days, followed by measuring the mRNA levels for Hh signal‐specific markers (Ptch1, Gli1, Gli2, and Gli3) using RT‐qPCR analysis (Figure 5d).^[^ ^33^ ^]^ In the HhAg group, the mRNA level of Ptch1 was ca. 1.3‐fold higher compared to the control group, and GLI‐Kruppel family members Gli1, Gli2, and Gli3 were elevated by ca. 7.0, 1.6, and 1.6‐fold, respectively. This trend was consistent with the initiation of Hh signaling (Figure 5e): Hh signaling is initiated when the Hh ligand binds to Patch1, followed by relieving the inhibition of Smo, thereby activating downstream signal transduction through markers such as GLI‐Kruppel family members Gli1, Gli2, and Gli3.^[^ ^34^ ^]^ Mechanistic studies have demonstrated that Hh signaling is crucial for enthesis formation during development and is reactivated during rotator cuff healing.^[^ ^34^ ^]^ Activation of Hh signaling at the injured enthesis promotes fibrocartilage regeneration and mineralization.^[^ ^35^ ^]^ However, it remains unclear how BMP, Wnt, and Hh signaling pathways combine to specifically regulate enthesis development.^[^ ^34^ ^]^

To further distinguish the effects of scaffold architecture and effector distribution, we incorporated funnel‐shaped microchannels into the scaffold containing constant concentrations (i.e., non‐graded) of HAp and HhAg. Specifically, we cultured hMSCs on the scaffolds with constant levels of HAp, HhAg, or both for 21 days, followed by staining cells with actin, DAPI, and lineage‐specific markers including OPN (osteogenic), collagen type X (Col‐10, hypertrophic chondrocyte), and scleraxis A (SCXA, tenogenic). As shown in Figures S7–S9 (Supporting Information), for the HAp‐only group, most cells expressed OPN throughout the microchannels, while only a few cells showed detectable levels of SCXA or Col‐10. In contrast, the cells in the HhAg‐only scaffold showed predominant expression of SCXA and/or Col‐10, with minimal OPN detection. The scaffold group with constant HAp and HhAg concentrations exhibited mixed cell phenotypes, with some cells expressing OPN and others expressing SCXA or Col‐10. Most importantly, in all instances, no gradient was observed along the vertical direction of the scaffold. The ability of HAp and HhAg to induce distinct lineage‐specific differentiation provides a key prerequisite for achieving cell‐graded differentiation within a dual‐graded scaffold system.

### 2.5 Graded Differentiation of hMSCs in the Scaffold

**Figure 6:**
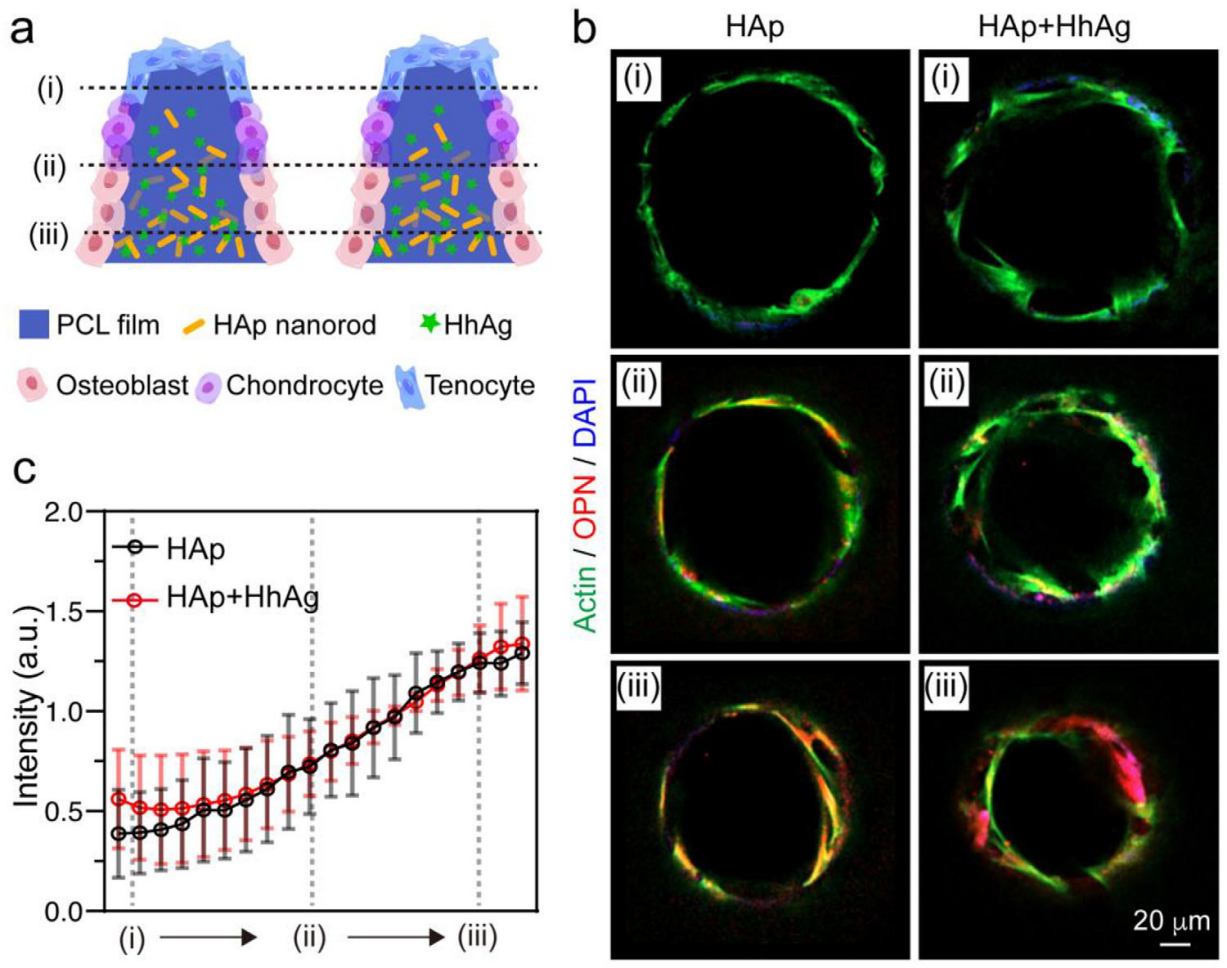
Osteogenic differentiation of hMSCs seeded in the scaffolds after 21 days of culture. a) Schematic illustrating different sections of the scaffold. b) Fluorescence images of the scaffolds after actin (green), OPN (red), and DAPI (blue) staining of the hMSCs cultured for 21 days. c) Plots of the fluorescence intensity when moving from section (i) to section (iii). Twelve microchannels from three individual scaffolds were analyzed. Data in c) are presented as mean ± SD.

Confirmation of the individual differentiation‐promoting effects of HAp and HhAg establishes the groundwork for examining their combined impact in the dual‐graded scaffolds to drive graded differentiation of hMSCs. Specifically, we seeded hMSCs into the scaffolds and then cultured for 21 days, followed by staining the cells with actin, OPN, and DAPI and visualization using confocal microscopy. As depicted in **Figure** **6a**, we again focused on three sections along the vertical direction of each scaffold. Notably, for both scaffolds, the expression of OPN was higher in the bottom section than in the other two (Figure 6b). Fluorescence intensity changes for OPN and DAPI along the microchannels confirmed progressively enhanced osteogenic differentiation with increasing HAp nanorods content in both scaffolds (Figure 6c). No significant changes were observed with the addition of HhAg. It is important to note that while the fluorescence intensity varied among different scaffold pieces within the same group, they consistently exhibited higher OPN expression toward the bottom (Figure S10, Supporting Information). Taken together, increasing HAp content along the scaffold's vertical direction led to enhanced osteogenic differentiation of stem cells at the bottom of both types of scaffolds.

**Figure 7:**
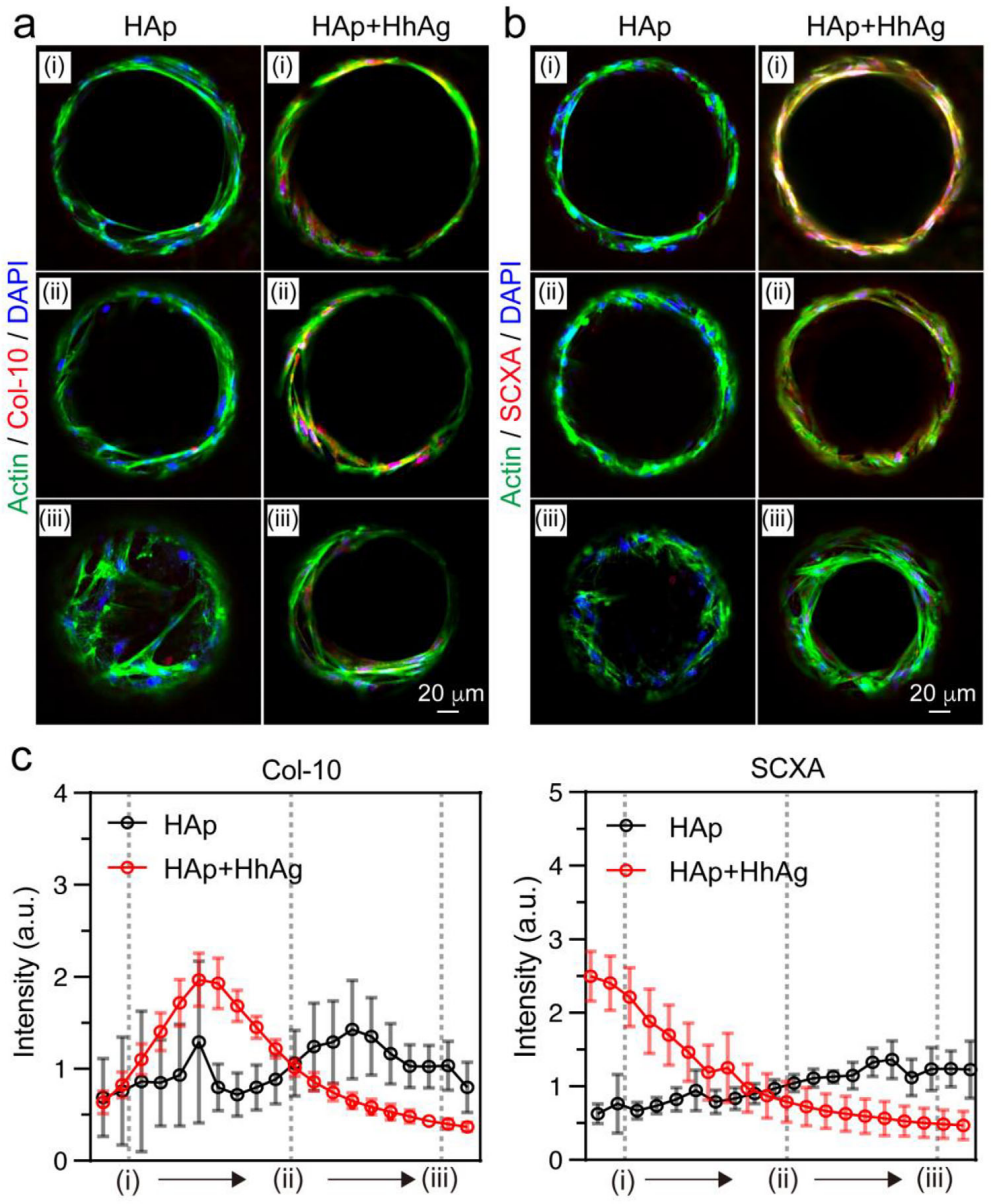
Cell chondrogenic and tenogenic differentiation of the hMSCs seeded in the biomimetic scaffolds and then cultured for 21 days. a,b) Fluorescence images of the scaffolds after actin (green), Col‐10 or SCXA (red), and DAPI (blue) staining of the hMSCs cultured for 21 days. c) Plots of the fluorescence intensity when moving from section (i) to section (iii). Twelve microchannels from three individual scaffolds were analyzed. Data in c) are presented as mean ± SD.

Immunofluorescence staining for the tenocyte‐specific marker SCXA and the hypertrophic chondrocyte marker Col‐10 after 21 days of cell differentiation revealed a graded distribution of these cell types.^[^ ^36^ ^]^ Again, the sections of interest correspond to those marked in Figure 6a. As shown in  Figure **7a,b**, in the absence of the HhAg gradient, there was no significant expression of SCXA and Col‐10 among any of the three sections of the HAp‐graded scaffold. In contrast, SCXA expression in the top section of the dual‐graded scaffold was higher than that in the other two sections, showing a decreasing trend from the top to the bottom. Through semi‐quantification of the fluorescence intensities of Col‐10, SCXA, and DAPI along the funnel‐shaped microchannels, we found that the intensity of Col‐10 initially increased before decreasing, whereas the intensity of SCXA progressively decreased (Figure 7c). Similar to the changes in OPN expression described above (Figure 6), these trends remained highly consistent across scaffold pieces within the same group (Figure S11, Supporting Information). The data demonstrated a transition from tenocytes to chondrocytes along the vertical direction, which is closely correlated with the HhAg‐induced signaling transduction in tendon enthesis formation. Combining the gradient of osteogenic differentiation of cells induced by HAp nanorods in the scaffold discussed in Figure 6, the dual‐graded scaffold fully recreated the transition of cell phenotype from tenocytes to chondrocytes and osteoblasts, providing new potential for tendon‐to‐bone repair/regeneration.

In the present work, we focused on the rational design, fabrication, and in vitro evaluation of the dual‐graded scaffolds for guiding graded cell differentiation. However, the effectiveness of these scaffolds in vivo is yet to be validated. Moving forward, the scaffolds can be tailored into disk‐shaped implants of various sizes to fit the defect site between the tendon and the bone. Using the rabbit rotator cuff model as an example, the scaffolds can be cut to 5 mm in diameter and 270 µm in thickness to match the dimensions of the tendon‐to‐bone attachment footprint. In addition, since the presence of microchannel structures throughout the scaffold may facilitate angiogenesis and enhance the survival of implanted cells under physiological conditions,^[^ ^37^ ^]^ it is of great interest to investigate the role of the funnel‐shaped channel architecture in promoting neovascularization and supporting cell viability in vivo. Furthermore, several important aspects warrant further investigation, including scaffold‐tissue integration, signaling crosstalk arising from the dual‐effectors, and the structural evolution of the HAp component during the healing process. A deeper understanding of these factors will be critical for advancing the clinical translation of the proposed scaffold platform.

## 3 Conclusion

We have demonstrated the fabrication of a biomimetic scaffold featuring dual gradients of HAp nanorods and HhAg, together with an array of funnel‐shaped microchannels, to regulate the graded differentiation of stem cells for tendon‐to‐bone repair. Within a period of 24 h, hMSCs successfully infiltrated, adhered, and proliferated along the microchannel walls of the scaffold. More significantly, the gradients in osteogenic effector HAp and enthesis fibrocartilage effector HhAg synergistically induced the graded differentiation of hMSCs into osteoblasts, chondrocytes, and tenocytes. Altogether, the gradients created in the biomimetic scaffold using a simple diffusion method promoted the simultaneous and uninterrupted multiple paths for the differentiation of stem cells, allowing for a seamless transition in cell phenotype to match the native tendon enthesis. This work holds promise for the repair/regeneration of connective tissue interfaces like the tendon enthesis.

## 4 Experimental Section

### Chemicals and Materials

Ca(NO_3_)_2_·4H_2_O, oleic acid, Na_3_PO_4_ 12H_2_O, 1‐octadecylamine, PCL (Mn ≈ 80000), 1,4‐dioxane, triton X‐100, poly‐l‐lysine, RhB, alizarin red, and bovine serum albumin (BSA) were obtained from Sigma‐Aldrich. α‐Minimum essential medium (α‐MEM), fetal bovine serum (FBS), and penicillin/streptomycin were purchased from Gibco. HhAg was obtained from Cellagen Technology. CCK8 kit was purchased from Dojindo. Phosphate‐buffered saline (PBS), calcein AM, and ethidium homodimer‐1 (EthD‐1) were ordered from Thermo Fisher. Anti‐Col‐10 (mouse monoclonal, ab49945, 1:500) and goat anti‐rabbit IgG H&L Alexa Fluor 594 (ab150080, 1:1000) were purchased from Abcam. Anti‐OPN (rabbit polyclonal, 22952‐1‐AP, 1:200) and CoraLitePlus 488‐Phalloidin (1:200, PF00001) were ordered from Proteintech. Anti‐SCXA (rabbit polyclonal, PA5‐23943, 1:500), goat anti‐rabbit IgG H&L Alexa Fluor 488 (A‐11012, 1:1000), and goat anti‐mouse IgG H&L Alexa Fluor 488 (A‐11011, 1:1000) was ordered from Invitrogen.

### Synthesis of HAp Nanorods

HAp nanorods were prepared using a hydrothermal method.^[^ ^6^, ^11^ ^]^ Specifically, the aqueous solution of Ca(NO_3_)_2_ (0.28 m, 7 mL) and Na_3_PO_4_ (0.168 m, 7 mL) was sequentially added to 20 mL of anhydrous ethanol and then stirred at 300 rpm for 20 min. The mixture was transferred into a Teflon‐lined autoclave and heated at 140 °C for 12 h. Afterward, the nanorods were collected by centrifugation (8000 rpm, 10 min) with cyclohexane and anhydrous ethanol for further use.

### Fabrication of Biomimetic Scaffold

We fabricated the biomimetic scaffold through a diffusion method. First, 0.6 g of PCL, 0.6 g of HAp, and 100 nm HhAg were uniformly dissolved in 12 mL of 1,4‐dioxane and then transferred into a 10 cm glass dish. Afterward, the dish was placed in a fume hood overnight to allow solvent evaporation slowly to obtain PCL‐HAp‐HhAg film. Then, we cast the PCL solution (0.6 g, 12 mL) onto the PCL‐HAp‐HhAg film, followed by evaporating overnight to obtain the dual‐graded film. The preparation of HAp‐graded film excluded HhAg from the above synthesis process. In this case, we replaced HhAg with RhB (200 µL, 20 mg mL^−1^) to obtain a HAp+RhB graded film. Afterward, a femtosecond laser (OPTEC WS‐Flex) was employed to drill a 10× 10 array of microchannels for scaffolds, with the gradient film attached to a glass slide and placed inside a controlled chamber. The laser was set to operate at 4 kHz, 18 W power, and 100 mm s^−1^ speed. For comparison, scaffolds with constant concentrations of HAp, HhAg, or both were prepared by dissolving 1.2 g of PCL, 0.6 g of HAp, and/or 100 nm of HhAg in 24 mL of 1,4‐dioxane. The resulting solution was then transferred into a 10‐cm glass dish and allowed to evaporate overnight to create uniform films. The films were subsequently processed using the same laser‐drilling protocol to create microchannels. The concentrations of HAp and HhAg were chosen to represent the levels anticipated in the middle region of a gradient scaffold.

### Local Modulus Measurement with Nanoindenter

The scaffold was cross‐sectioned into 50 µm‐thick slices and the local modulus along the vertical axis was measured using a nanoindenter (TriboIndenter 980, Bruker) equipped with a Berkovich three‐sided pyramidal tip (Tip radius: ≈200 nm). Indentation tests were performed under a load‐controlled mode with a peak force of 500 µN and an average area of 1.5 × 10^7^ nm^2^. Using the Piezo automation function, eight regions (10 µm × 10 µm, n = 5 per region) along the vertical axis were tested to determine the reduced elastic modulus (E^*^). According to the Oliver‐Pharr method ($\frac{1}{E^{*}}=\frac{1-\nu_{1}^{2}}{E_{1}}+\frac{1-\nu_{2}^{2}}{E_{2}}$), the Young's modulus of the sample (E_1_) was calculated. Here, the ν_2_ and E_2_ represent the Poisson's ratio (0.07) and elastic modulus (1140 GPa) of the nanoindenter tip, respectively. The Poisson's ratio of the sample was assumed to be isotropic at 0.3.^[^ ^38^ ^]^

### Instrumental Characterization

The morphology and physical properties of HAp nanorods were analyzed using HT7700 TEM (Hitachi) and Miniflex600 (Rigaku). The structure of the biomimetic scaffold was characterized by SEM (Hitachi SU8230). To examine the graded distribution of HAp and HhAg along the vertical direction, the scaffold was cryo‐sectioned into 50 µm‐thick slices and analyzed using the EDX mode of SEM, Raman microscopy, and ToF‐SIMS. In addition, the distribution of RhB in the HAp+RhB film was observed using the Z‐stack mode of a confocal microscope (Zeiss LSM 900).

### Cell Seeding and Culture

hMSCs were obtained from a commercial source (Lonza, Basel, Switzerland) and were recovered from cryopreservation. The cells were cultured until the third generation in α‐MEM supplemented with 10% FBS and 1% penicillin/streptomycin. The scaffolds were treated in a plasma cleaner (Plasma Etch PE50) for 2 min to enhance their surface hydrophilicity and improve cell attachment onto the scaffold. After being sterilized in 75% ethanol overnight, the scaffolds were immersed in 10 µg/mL of poly‐l‐lysine, shaken on a shaker platform (Corning GyroTwister S1000‐40) at 50 rpm overnight, and then immersed in a fresh cell culture medium overnight. Subsequently, hMSCs were seeded at a density of 10^4^ cells mL^−1^ into the scaffold and cultured at 37 °C in a humidified chamber containing 5% CO_2_. The culture medium was replaced every other day.

### Cell Live/Dead Staining

Cell survival was assessed using calcein AM and EthD‐1 for live/dead staining. Cells were allowed to adhere for 24 h, after which the culture medium was replaced with serum‐free α‐MEM containing 5 µm calcein AM and 4 µm EthD‐1, and the cells were incubated at 37 °C in a humidified chamber containing 5% CO_2_ for 30 min. Following three washes with PBS, the samples were observed under a confocal microscope (Zeiss LSM 900).

### CCK8 Assay

After culturing cells with scaffolds for 1, 2, and 3 days, the cell culture medium was replaced with serum‐free α‐MEM containing 10% CCK8 solution and cultured at 37 °C for 1 h. Afterward, the cell culture medium was collected, and its absorbance at 492 nm was measured using a microplate reader (Infinite 200, TECAN). To compare cell viability across different regions (top, middle, and bottom), the scaffold was cross‐sectioned into three 50 µm‐thick slices before cell seeding, without altering any other experimental parameters.

### Cell SEM

After culturing cells with scaffolds for 24 h, cell‐seeded scaffolds were fixed in 2.5% glutaraldehyde solution for 30 min. Afterward, the samples were dehydrated with graded ethanol of 30%, 50%, 70%, 80%, 90%, and 100%. Finally, the samples were observed under SEM.

### RT‐qPCR Analysis

Cells were cultured on the plain PCL film or PCL film containing a constant concentration of HAp nanorods/HhAg in 6‐well plates for 14 days. The cells were then treated with 1 mL of TRIzol reagent to extract total RNA. The purity and concentration of the RNA were assessed using a spectrophotometer (ThermoFisher Nanodrop). Next, reverse transcription of the total RNA into cDNA was performed according to the instructions provided with the QuantiTect Reverse Transcription Kit. We then utilized the StepOnePlus Real‐Time PCR System to obtain the C_T_ value. The relative mRNA expression level for each target gene was calculated as 2^−ΔΔCt^, with β‐actin serving as the housekeeping gene. The primer sequences are listed in Table S1 (Supporting Information).

### Immunofluorescence

After 21 days of culture, cell‐seeded scaffolds were fixed with 4% paraformaldehyde for 20 min and then immersed in 0.1% Triton X‐100 for 5 min. Subsequently, the samples were blocked with 1% BSA for 30 min and then incubated with the primary antibody overnight. Afterward, the samples were incubated with a secondary antibody for 1 h at room temperature in the dark to bind to the primary antibody. Then, the samples were incubated with CoraLite Plus 488‐Phalloidin (1:200) for 20 min and DAPI for 5 min. Finally, the samples were observed using the confocal microscope (Zeiss LSM 900). We provided the concentration and catalog numbers of antibodies in the section on chemicals and materials.

### Alizarin Red Staining

After 21 days of culture, the cells were fixed with 4% paraformaldehyde for 15 min and then washed with PBS three times. Afterward, the samples were stained with the alizarin red solution for 10 min and captured using a smartphone camera.

### Statistical Analysis

Statistical analyses were performed with GraphPad Prism 10.0, and the results were presented as the mean ± SD. Statistical significance was determined using a two‐tailed unpaired Student's t‐test. The fluorescence intensities were analyzed using Zen (blue) software from Zeiss.

## Conflict of Interest

The authors declare no conflict of interest.

## Supporting information



Supporting Information

## Data Availability

The data that support the findings of this study are available from the corresponding author upon reasonable request.
