# Supplementary material for: Biomimetic Scaffolds with Dual Gradients of Biological Effectors for Tendon‐to‐Bone Repair
Source: Adv Healthc Mater. 2025 Jul 15;14(28):2503171. doi: 10.1002/adhm.202503171 (PMC12494179; doi:10.1002/adhm.202503171)
Supplement: Supplementary file 1 — Supporting Information [file ADHM-14-0-s001.docx]

**Supporting Information**

**Biomimetic Scaffolds with Dual Gradients of Biological** **Effectors for Tendon-to-Bone Repair**

Min Hao,^#^ Yidan Chen,^#^ Stavros Thomopoulos, and Younan Xia*

Prof. Y. Xia, Dr. M. Hao

The Wallace H. Coulter Department of Biomedical Engineering

Georgia Institute of Technology and Emory University

Atlanta, GA 30332 (USA)

E-mail: [younan.xia@bme.gatech.edu](mailto:younan.xia@bme.gatech.edu)

Y. Chen

School of Materials Science and Engineering

Georgia Institute of Technology

Atlanta, GA 30332 (USA)

Prof. Y. Xia

School of Chemistry and Biochemistry

Georgia Institute of Technology

Atlanta, GA 30332 (USA)

Prof. S. Thomopoulos

Department of Orthopedic Surgery and Department of Biomedical Engineering

Columbia University

New York, NY 10032 (USA)

^#^ These authors contributed equally to this article.


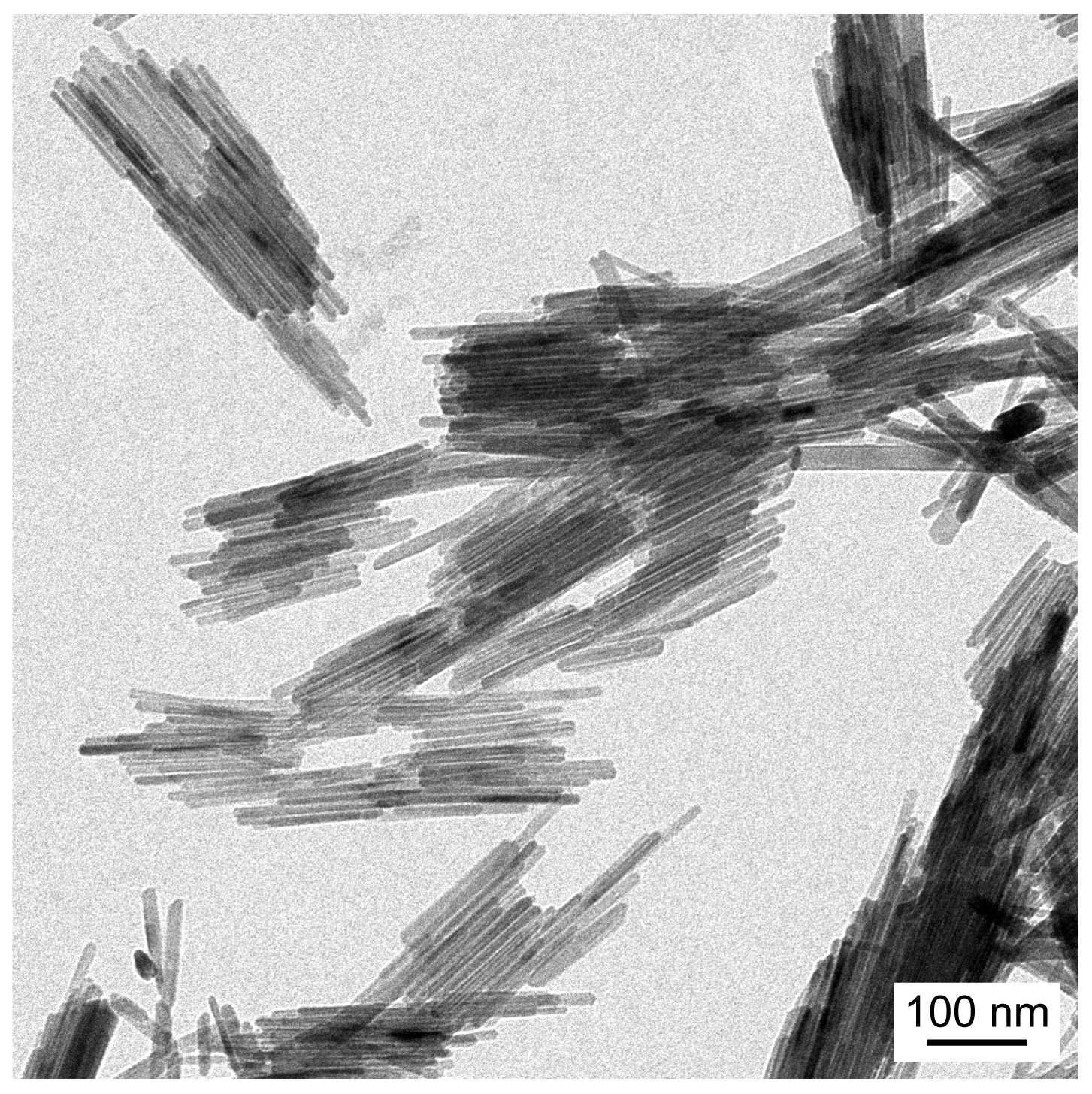


**Figure S1.** TEM image of HAp nanorods obtained after embedding in PCL matrix for 3 days, followed by dissolution of PCL with 1,4-dioxane and centrifugation.


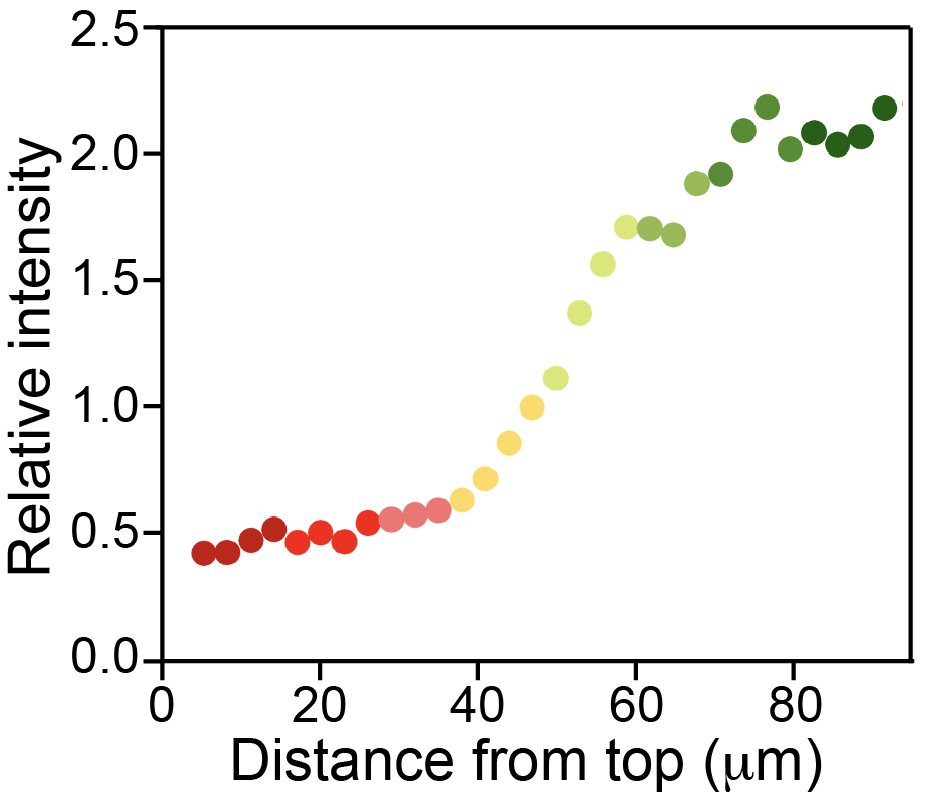


**Figure S2.** Ratio between the intensities of Raman peaks at 960 cm^-1^ (P-O stretching) and 1724 cm^-1^ (C=O stretching) in the graded region.


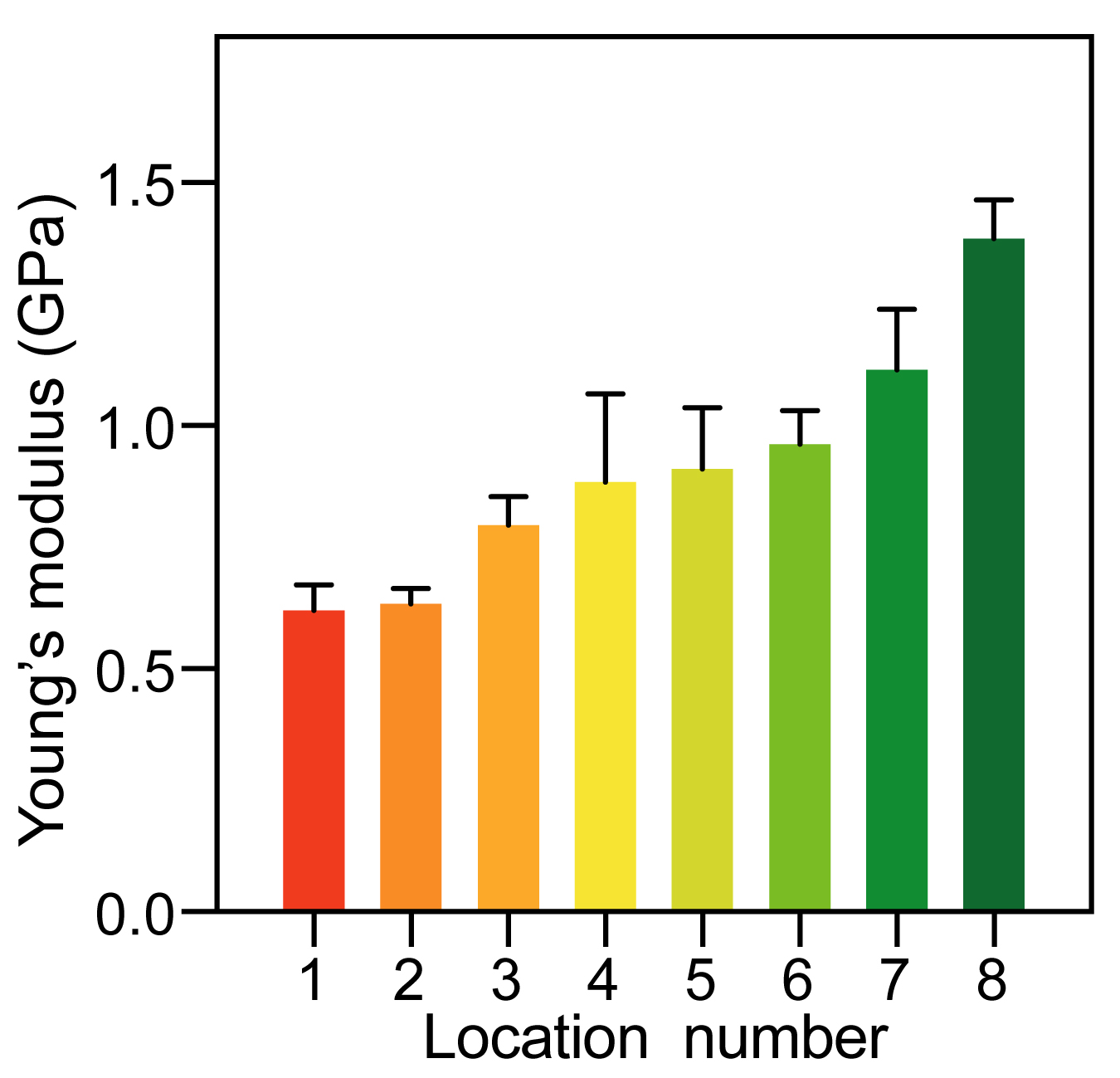


**Figure S3.** Local Young’s modulus across the cross-section of the HAp+HhAg scaffold, from the PCL-rich region (location 1) to the HAp+HhAg-rich region (location 8) across a distance of about 70 μm (n=5).


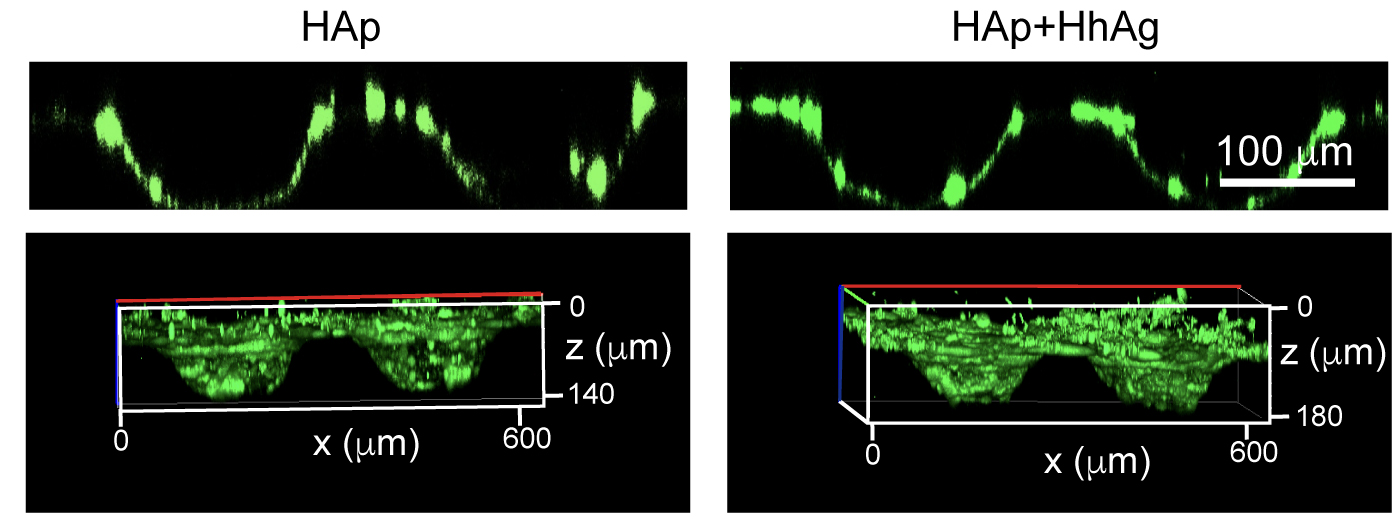


**Figure S4.** The cross-sectional and three-dimensional Z-stack fluorescence micrographs of the hMSCs seeded in the scaffolds and then cultured for 24 h, followed by live/dead staining. The live and dead cells were stained green and red, respectively.


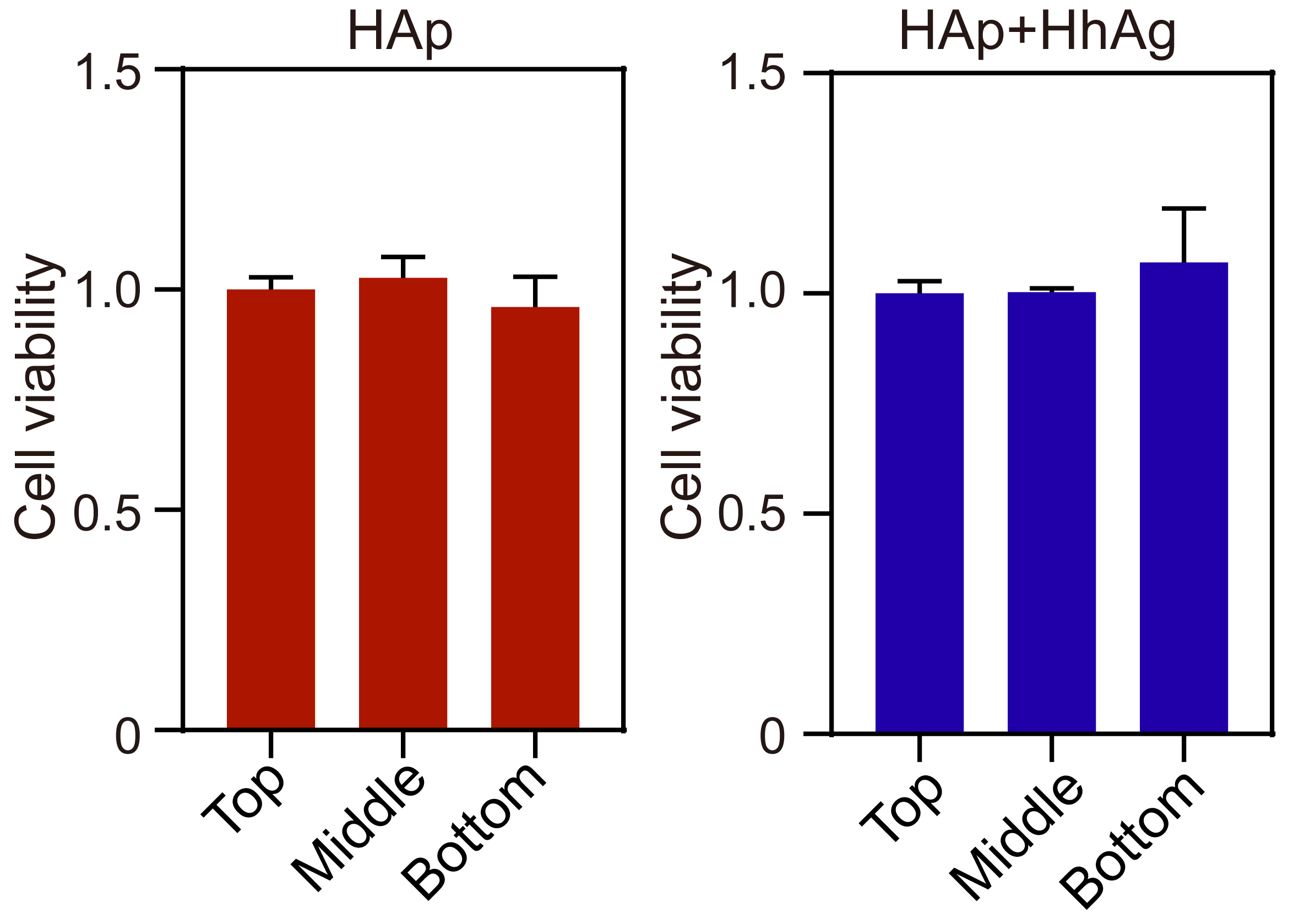


**Figure S5.** CCK8 assay of the hMSCs after culture with the cryo-sectioned sections (top, middle, bottom) of scaffolds for 24 h. The data are normalized to the top section and presented as mean ± standard deviation (SD) (N=3).


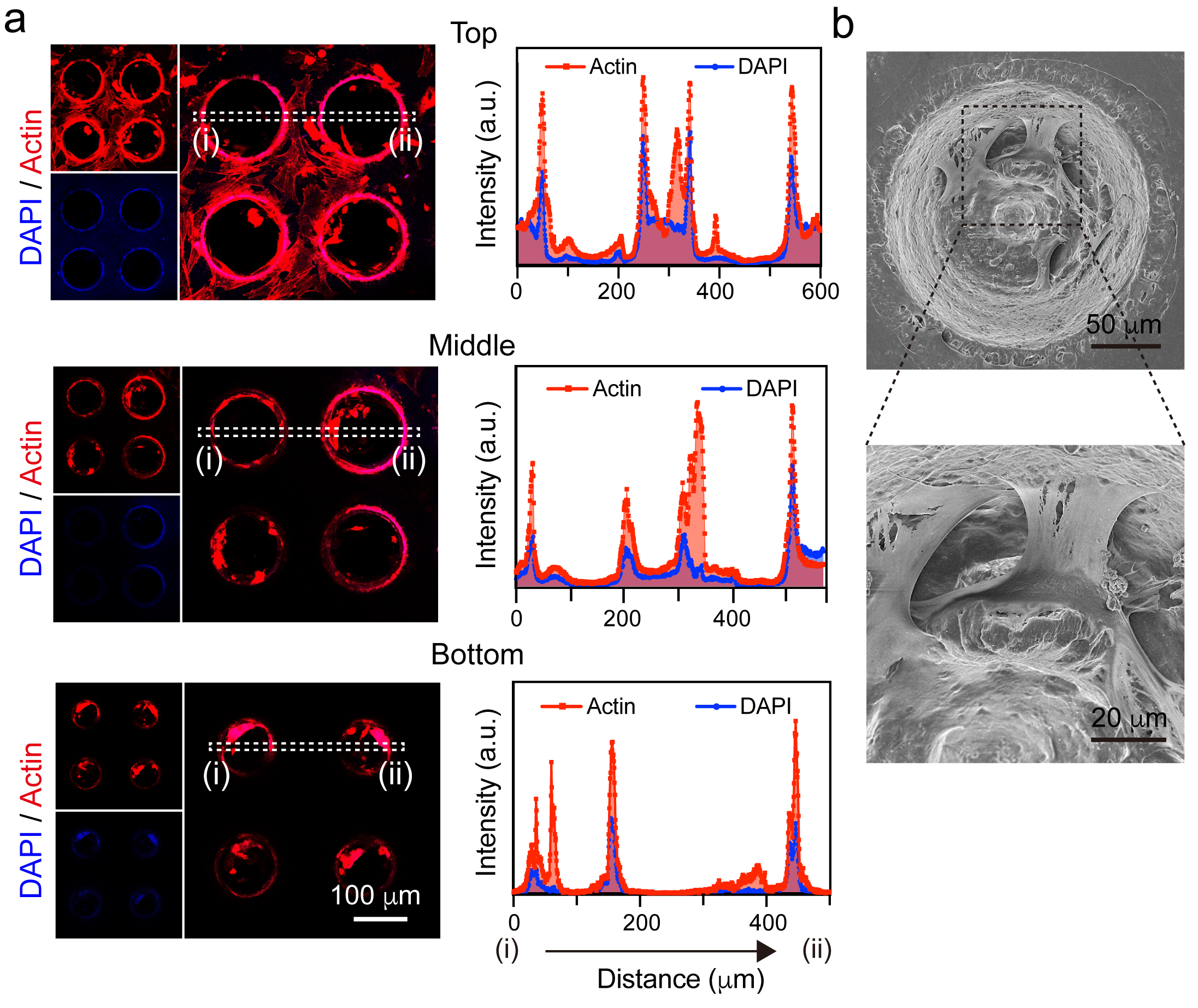


**Figure S6.** Distribution and morphology of the cells cultured in the HAp-graded scaffolds. (a) Fluorescence micrographs (left) and fluorescence intensity distributions (right) of the cells after culture with the scaffold for 24 h, followed by actin (red) and DAPI (blue) staining. The plots of fluorescence intensity correspond to the regions marked by boxes on the left. (b) SEM images of the scaffold after being seeded with hMSCs and then cultured for 24 h.


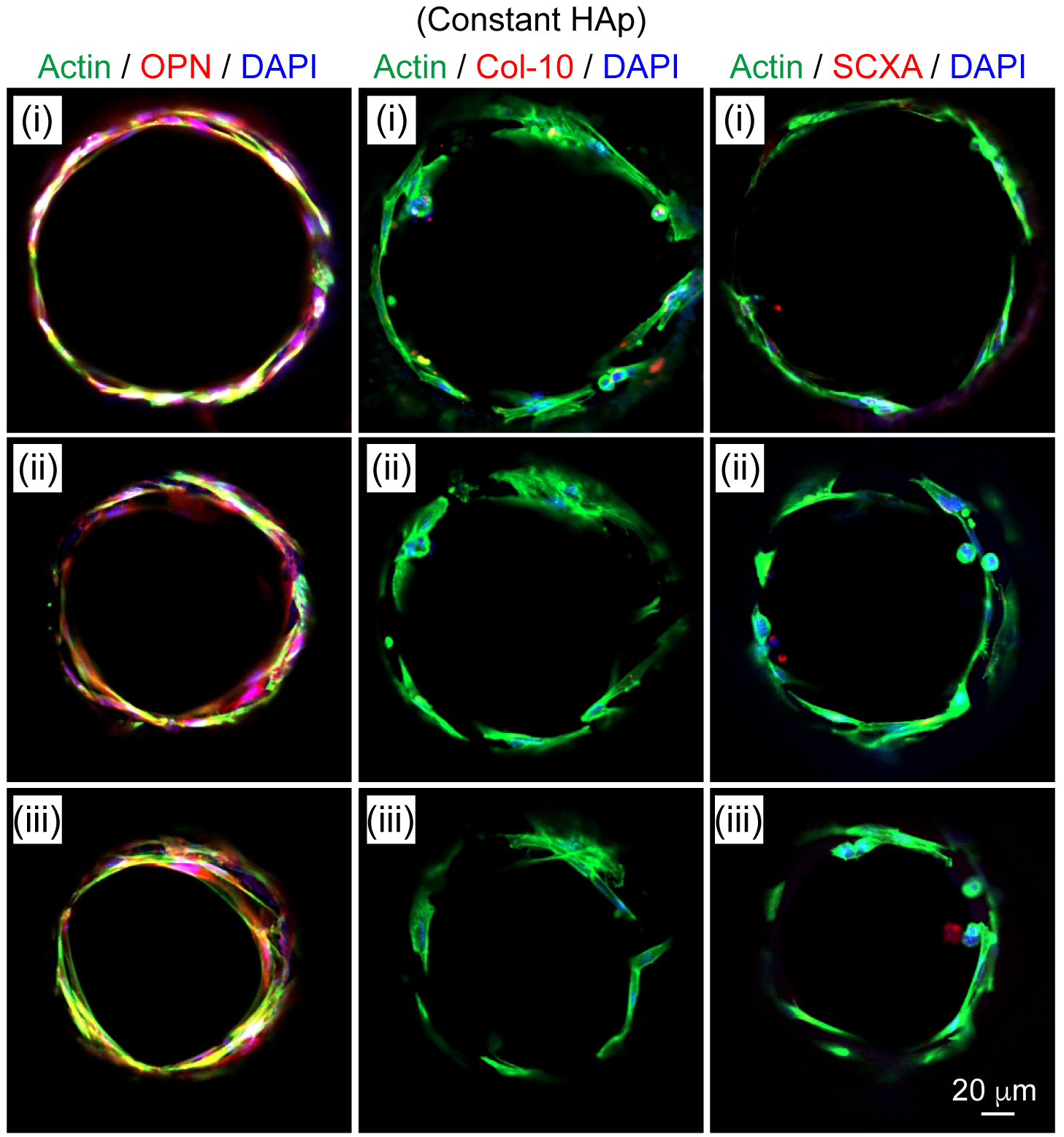


**Figure S7.** Fluorescence images of the scaffolds with a constant concentration of HAp after actin (green), OPN/Col-10/SCXA (red), and DAPI (blue) staining of the hMSCs cultured for 21 days. The labels (i), (ii), and (iii) indicate the top, middle, and bottom sections of the scaffolds. The sections of interest correspond to those marked in Figure 6a.


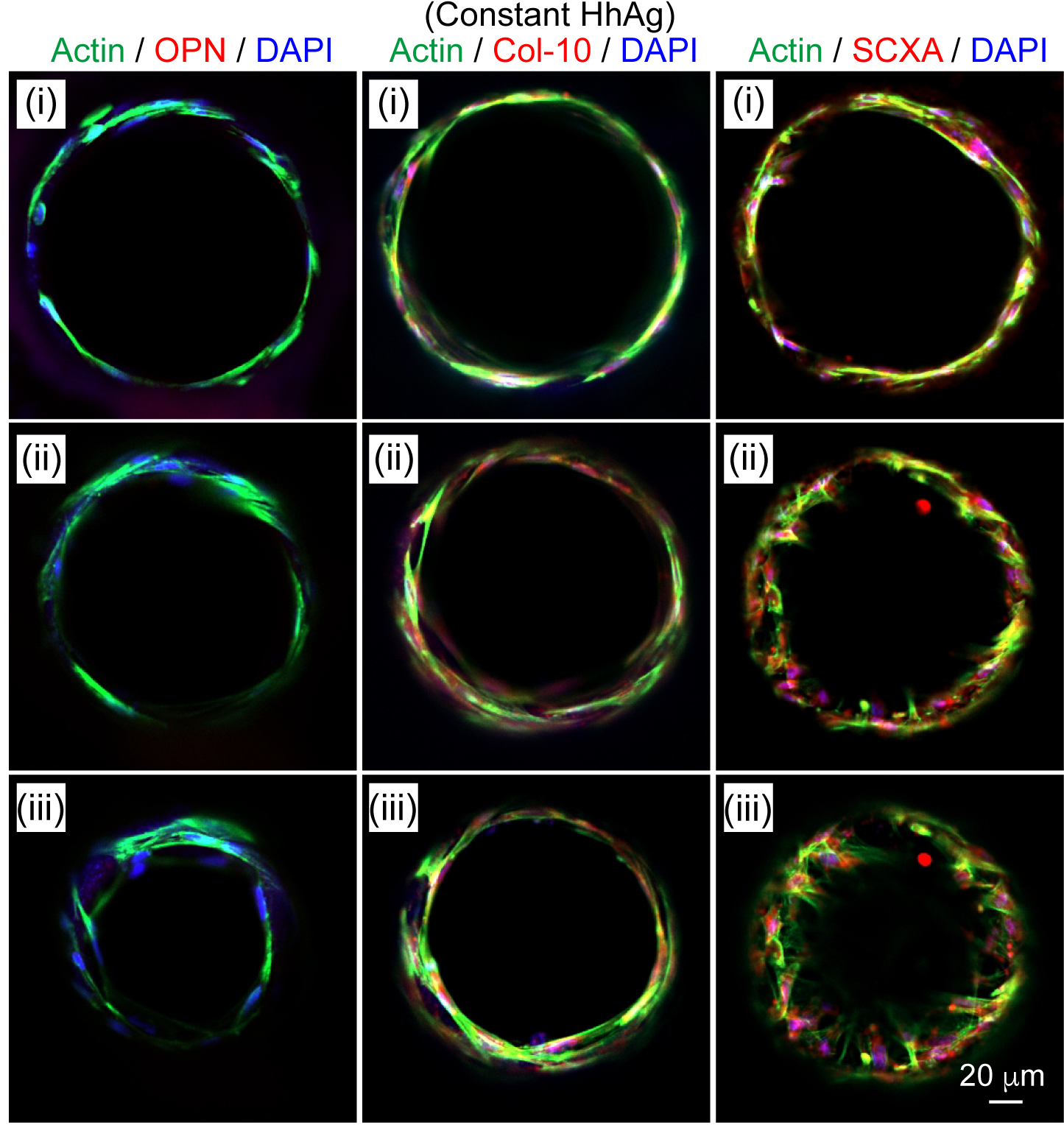


**Figure S8.** Fluorescence images of the scaffolds with a constant distribution of HhAg after actin (green), OPN/Col-10/SCXA (red), and DAPI (blue) staining of the hMSCs cultured for 21 days. The labels (i), (ii), and (iii) indicate the top, middle, and bottom sections of the scaffolds. The sections of interest correspond to those marked in Figure 6a.


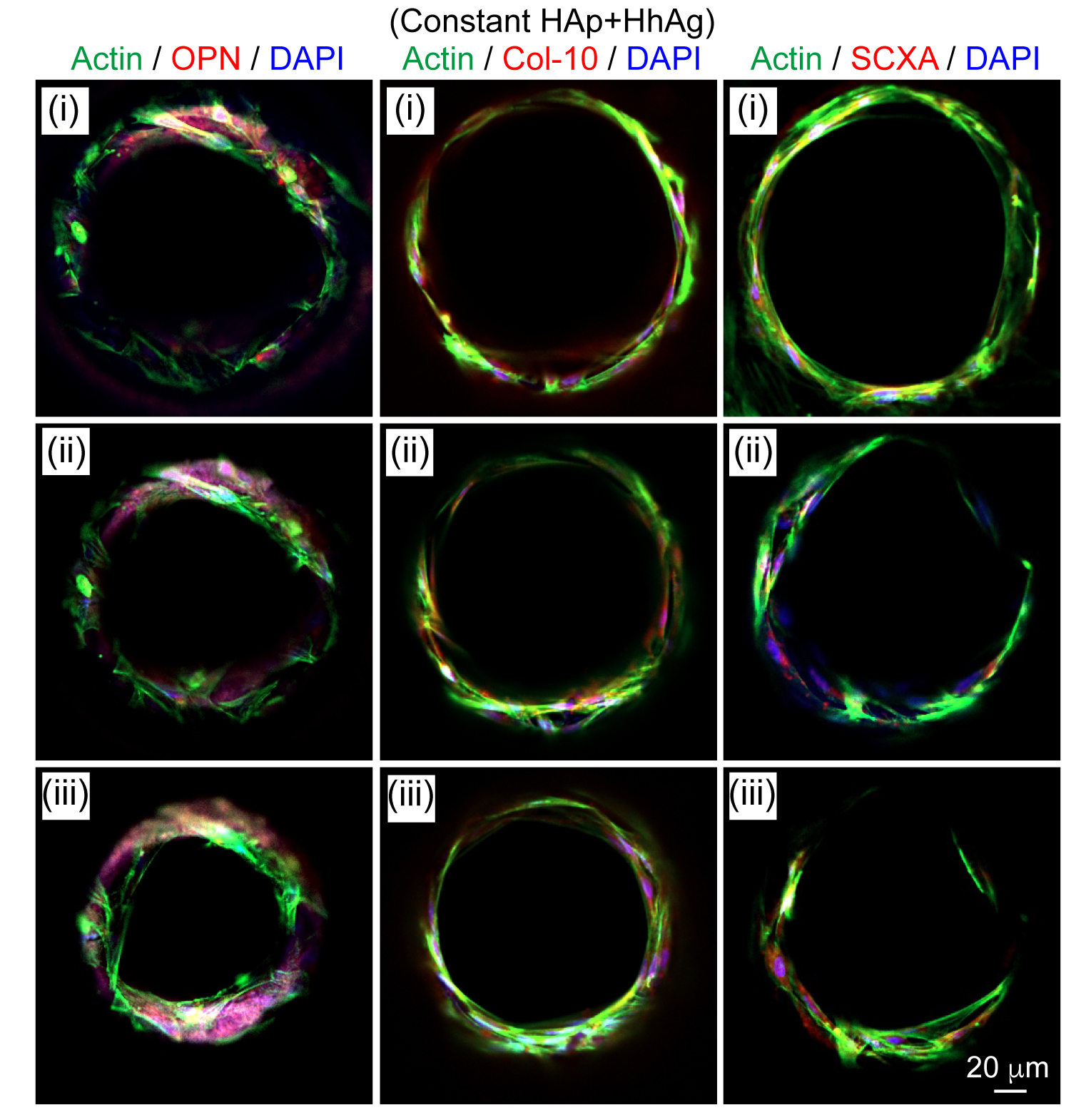


**Figure S9.** Fluorescence images of the scaffolds with a constant distribution of HAp+HhAg after actin (green), OPN/Col-10/SCXA (red), and DAPI (blue) staining of the hMSCs cultured for 21 days. The labels (i), (ii), and (iii) indicate the top, middle, and bottom sections of the scaffolds. The sections of interest correspond to those marked in Figure 6a.


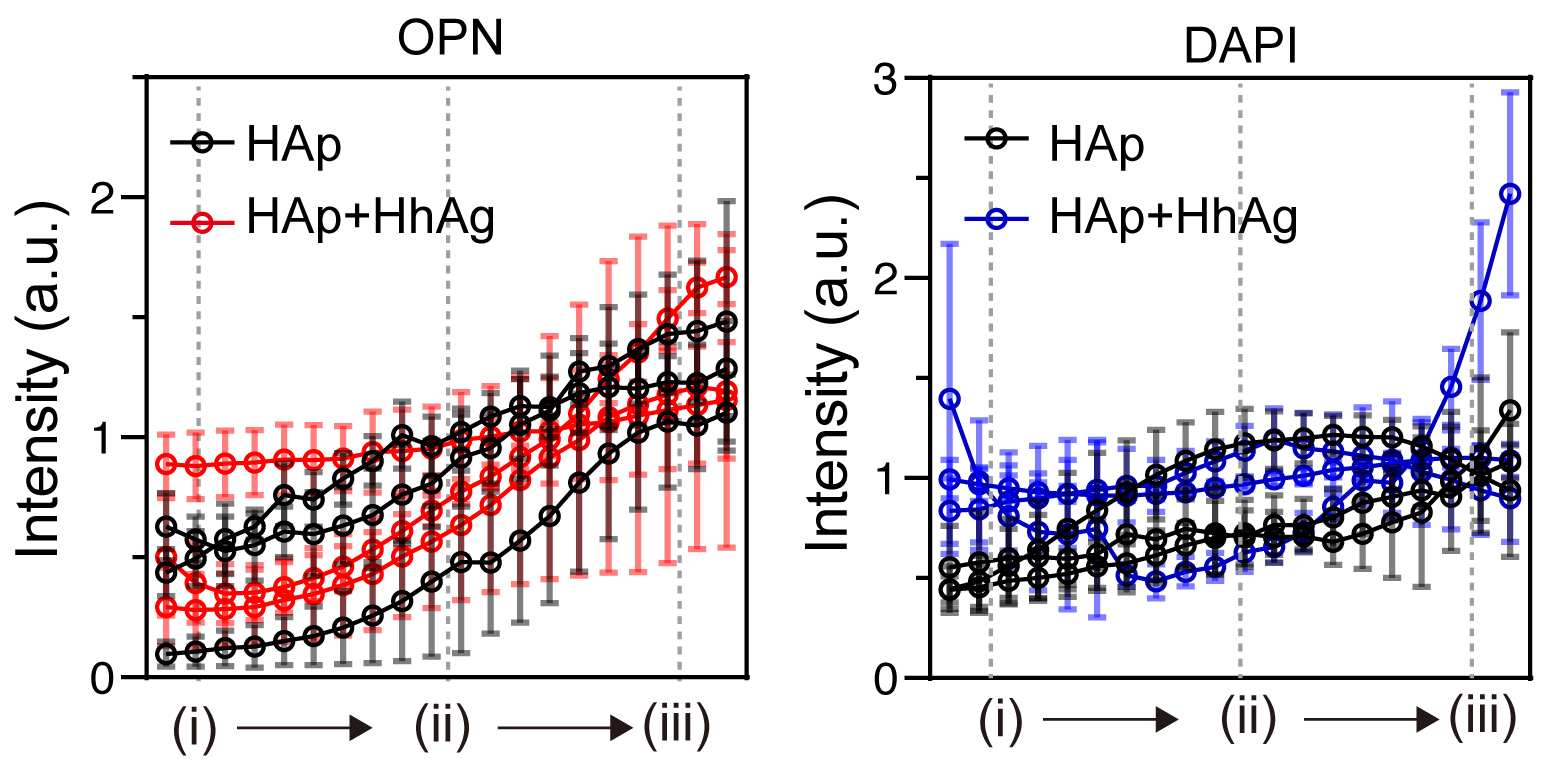


**Figure S10.** Plots of the fluorescence intensity of OPN and DAPI when moving from section (i) to section (iii). The sections of interest correspond to those marked in Figure 6a. Each line represents data obtained from a single scaffold containing four microchannels. Three independent scaffolds were included in the analysis of each group. Data are presented as mean ± SD.


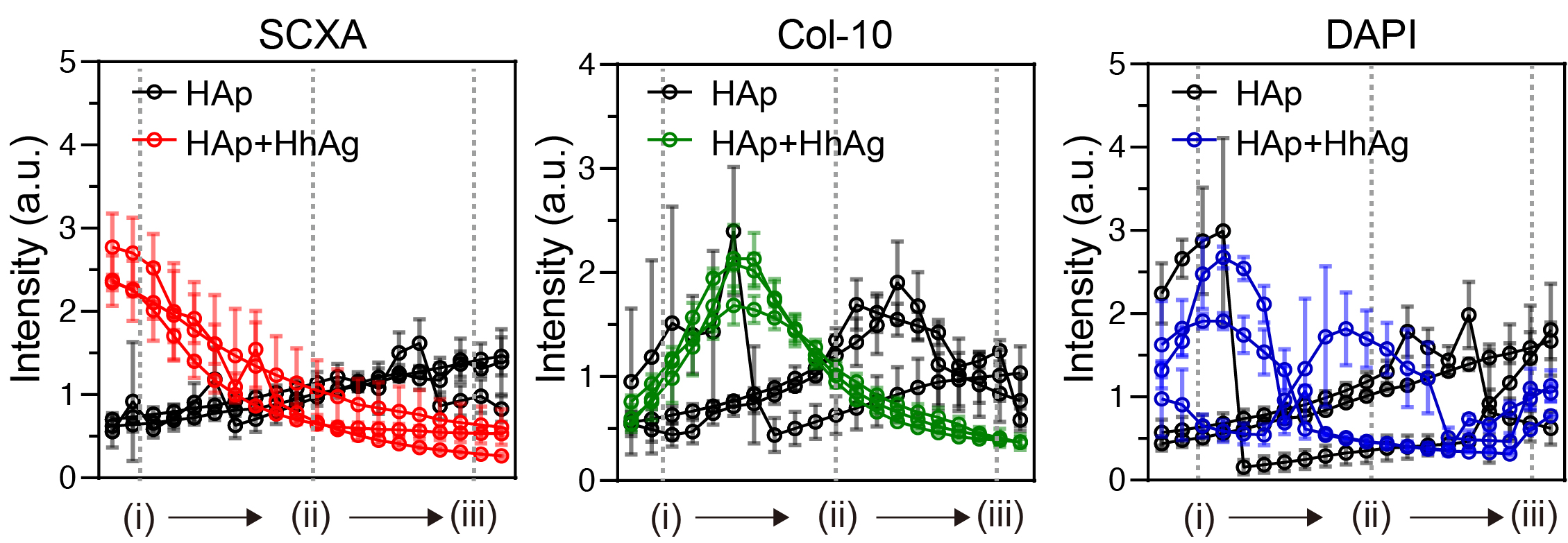


**Figure S11.** Plots of the fluorescence intensity of SCXA, Col-10, and DAPI when moving from section (i) to section (iii). The sections of interest correspond to those marked in Figure 6a. Each line represents data obtained from a single scaffold containing four microchannels. Three independent scaffolds were included in the analysis of each group. Data are presented as mean ± SD.

**Table 1.** The sequences of primers for RT-qPCR.

| Gene | Forward primers (5’-3’) | Reverse primers (5’-3’) |
| --- | --- | --- |
| β-actin | CATGTACGTTGCTATCCAGGC | CTCCTTAATGTCACGCACGAT |
| OPN | CCTCCTAGGCATCACCTGTG | CCACACTATCACCTCGGCC |
| OCN | CTGTATCAATGGCTGGGAGC | GCCTGGAGAGGAGCAGAACT |
| Runx2 | AATGCCTCCGCTGTTATG | TTCTGTCTGTGCCTTCTTG |
| Bmp2 | ACCCTTTGTACGTGGACTTC | GTGGAGTTCAGATGATCAGC |
| Ptch1 | TGAATCAAGGAGCTGCTGCG | CCCGGACTCTGCTTTCTTGT |
| Gli1 | CAGACAGAGGCCCACTCTTTTC | GAGGTGCGGATAACCGTCTG |
| Gli2 | CCCTACCGATTGACATGCGA | GCCGGATCAAGGAGATGTCA |
| Gli3 | ATGGGAAGTTCGGGGACTTGA | TTTTCAGTGGTCGTGGAGCTG |
